# The Role of Botulinum Toxin Type-A in Spasticity: Research Trends from a Bibliometric Analysis

**DOI:** 10.3390/toxins16040184

**Published:** 2024-04-09

**Authors:** Salvatore Facciorusso, Stefania Spina, Alessandro Picelli, Alessio Baricich, Gerard E. Francisco, Franco Molteni, Jörg Wissel, Andrea Santamato

**Affiliations:** 1 Spasticity and Movement Disorders “ReSTaRt”, Unit Physical Medicine and Rehabilitation Section, Department of Medical and Surgical Sciences, University of Foggia, 71122 Foggia, Italy; s.facciorusso89@gmail.com (S.F.); andrea.santamato@unifg.it (A.S.); 2 Department of Medical and Surgical Specialties and Dentistry, University of Campania “Luigi Vanvitelli”, 80138 Naples, Italy; 3 Department of Neurosciences, Biomedicine and Movement Sciences, University of Verona, 37100 Verona, Italy; alessandro.picelli@univr.it; 4 Physical Medicine and Rehabilitation, Department of Health Sciences, Università del Piemonte Orientale, 28100 Novara, Italy; alessio.baricich@med.uniupo.it; 5 Department of Physical Medicine & Rehabilitation, University of Texas Health McGovern Medical School, Houston, TX 77030, USA; gerard.e.francisco@uth.tmc.edu; 6 Villa Beretta Rehabilitation Center, Valduce Hospital Como, 23845 Costa Masnaga, Italy; franco56.molteni@gmail.com; 7 Department of Neurorehabilitation and Physical Therapy, Vivantes Hospital Spandau, 13585 Berlin, Germany; joerg@schwarz-wissel.de

**Keywords:** BoNT-A, spasticity, bibliometric analysis, research trends, neurological disorders

## Abstract

Botulinum toxin type-A (BoNT-A) has emerged as a key therapeutic agent for the management of spasticity. This paper presents a comprehensive bibliometric and visual analysis of research concerning BoNT-A treatment of spasticity to elucidate current trends and future directions in this research area. A search was conducted in the Web of Science database for articles focused on the use of BoNT-A in spasticity published between 2000 and 2022. We extracted various metrics, including counts of publications and contributions from different countries, institutions, authors, and journals. Analytical methods in CiteSpace were employed for the examination of co-citations, collaborations, and the co-occurrence of keywords. Our search yielded 1489 publications. Analysis revealed a consistent annual increase in research output. The United States, United Kingdom, and Italy were the leading contributors. The top institution in this research was Assistance Publique Hopitaux, Paris. The journal containing the highest number of relevant publications was *Toxins*. Key frequently occurring keywords were ‘stroke’, ‘cerebral palsy’, ‘adult spasticity’, and ‘upper extremity’. This study identified 12 clusters of keywords and 15 clusters of co-cited references, indicating the main focus areas and emerging themes in this field. This study comprehensively analyzed and summarized trends in BoNT-A research in the field of spasticity over the past 22 years.

## 1. Introduction

Spasticity is a disorder that typically develops as a result of lesions in the central sensorimotor network, leading to upper motor neuron syndrome [1]. It is characterized by a velocity-dependent increase in muscle tone and reflexes [2]. Its prevalence varies according to the underlying condition, with estimates indicating its occurrence in 25.3% to 39.5% of stroke survivors [3], up to 60% of multiple sclerosis patients [4], up to 30% of patients with traumatic brain injury [5], and more than 80% of the population with cerebral palsy [6]. Spasticity, aside from its cerebral and spinal causes, can also have a genetic basis, as evidenced in hereditary spastic paraplegias, which affect between two and five individuals per 100,000 worldwide [3]. This heterogeneity in prevalence, coupled with its impact on motor function and quality of life, underscores the challenges in managing spasticity effectively.

While spasticity may not always be debilitating, its severity often tends to increase over time, leading to significant changes in muscle structure [3,7]. This progression underscores the need for closer monitoring and multimodal interventions, especially in patients with moderate to severe paresis [8]. An international survey highlighted that 72% of patients with spasticity reported an impact on quality of life, with many also experiencing depression and loss of independence, reflecting the condition’s extensive impact not only on patients, but also on their families [9,10]. In line with this, non-motor symptoms, such as spasticity-related unpleasant sensations, can have a significant impact on daily activities [11].

Botulinum toxin type-A (BoNT-A) has emerged as a pivotal therapeutic agent in the management of spasticity owing to its ability to induce chemodenervation through its action on presynaptic neurons [12]. The use of BoNT-A has substantially increased over the years, demonstrating its growing acceptance and application in clinical practice. The evolution of BoNT-A as a treatment modality for spasticity reflects a significant shift in the approach to managing this condition. Initially, the focus was predominantly on the symptomatic relief of spasticity and related impairments [13,14,15]. However, with a growing body of evidence supporting the efficacy of BoNT-A in improving functional outcomes and quality of life, its use has expanded and has become more sophisticated [10,16,17].

Bibliometric analysis is an increasingly utilized approach in medical research for quantifying and characterizing scientific outputs [18]. It offers a systematic method to evaluate the impact, trends, and network of research on a given topic. Using tools such as CiteSpace, bibliometric studies can visualize the development and trajectory of a research area, including identifying key papers, authors, and institutions that have contributed significantly to the field [19].

In this study, we conducted a comprehensive bibliometric and visual analysis of the literature on the use of BoNT-A for spasticity from 2000 to 2022. Data were sourced from the Web of Science Core Collection (Clarivate). The primary objective was to map the evolution of BoNT-A research in the context of spasticity by elucidating key trends, major contributors, and emerging themes. This analysis aimed to provide a broad view of the global research landscape on this topic, highlighting the central role of BoNT-A in managing spasticity and guiding future research directions.

## 2. Results

### 2.1. Publication Outputs and Time Trend

**Figure 1:**
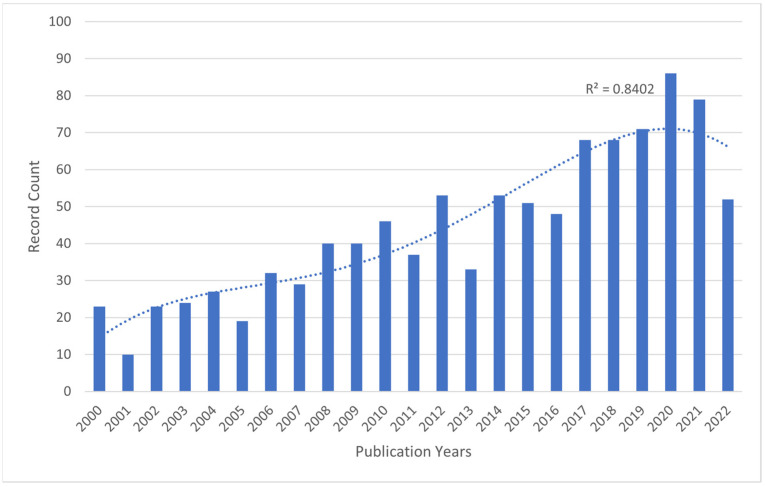
The number of publications related to BoNT-A and spasticity, extracted from the Web of Science Core Collection (WoSCC), 2000–2022. Bar chart showing number of publications per year and a dotted trendline displaying the increasing trend in record counts from 2000 to 2022.

In terms of publication output, 1489 papers on the use of BoNT-A for spasticity that were published between 2000 and 2022 were identified. Analyzing the publication trend, the data exhibited a progressive increase in research output, characterized by an initial gradual rise in the early 2000s, accelerating notably after 2007 (Figure 1). The period leading up to 2012 marked a phase of robust growth in publications. This trend continued with high output until 2016, after which a plateau was observed, albeit at elevated publication levels, until 2020.

The highest recorded number of publications on the topic was in 2020, with 127 records, accounting for 8.53% of the total 1489 records. This number dropped slightly by 2021, with 114 records (7.66% of the total). Overall, research on this topic has become more active over time, suggesting that the use of botulinum toxin for spasticity is a growing area of study.

### 2.2. Hot Topics in Literature Research

**Figure 2:**
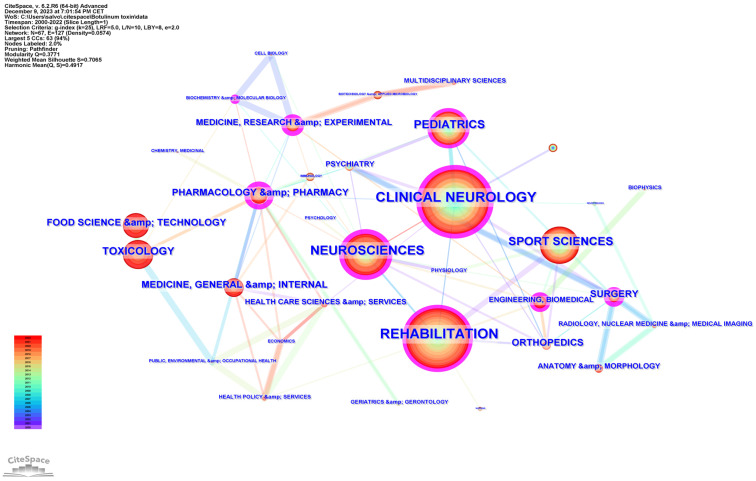
The research hotspot distribution related to BoNT-A and spasticity, extracted from the Web of Science Core Collection (WoSCC), 2000–2022.

Subject categories were extracted from Web of Science and mapped using CiteSpace. The generated graph showed 67 nodes, suggesting that the field of study involved 67 categories (Figure 2). The most frequent was “rehabilitation” (493 distributions), followed by “clinical neurology” (468 distributions), and “neuroscience” (251 distributions). The convergence of these disciplines underscores a multidisciplinary approach to understanding and treating neurological conditions, with rehabilitation serving as a crucial component of patient care and recovery.

Other frequent categories included “sport sciences” (210 distributions), “pediatrics” (181 distributions), and “toxicology” (107 distributions). The interconnectivity of nodes suggests a rich, collaborative research ecosystem geared towards advancing the understanding and treatment of spasticity.

### 2.3. Country Analysis

A total of 74 countries participated in the publication of studies on the use of BoNT-A and spasticity between 2000 and 2022. The top 10 most active countries are listed in Table 1. The United States contributed the most papers (359 publications, 24.11%), followed by the United Kingdom (170 publications, 11.42%), and Italy (169 publications, 11.35%).

**Figure 3:**
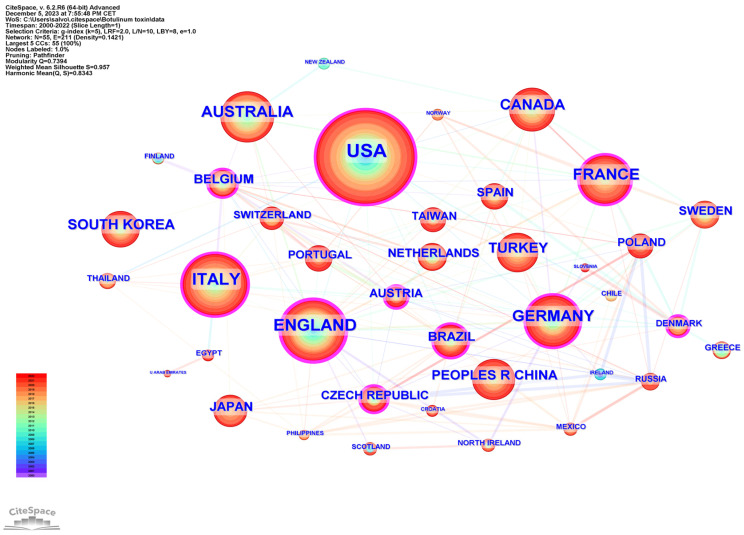
Network cooperation map of countries/regions. The nodes represent countries/regions, and the lines between the nodes represent cooperative relationships. The larger the node size, the larger the number of publications produced by that country. The nodes in the outermost area with purple rings indicate high centrality.

The international collaboration among countries is analyzed in Figure 3, generated by CiteSpace. The top three countries in terms of centrality (purple outer ring) were the United Kingdom (0.20), United States (0.15), and Italy (0.10).

### 2.4. Institution Analysis

A total of 2050 institutions, extracted from the author’s affiliations, published articles on the use of BoNT-A in spasticity over the 22 years analyzed. Table 2 lists the top 10 institutions, depending on the number of publications. Among these, the publications were derived from research institutes and universities. The three institutions with the highest number of publications were Assistance Publique Hopitaux Paris in France, UDICE-French Research Universities in France, and the University of London in the United Kingdom.

**Figure 4:**
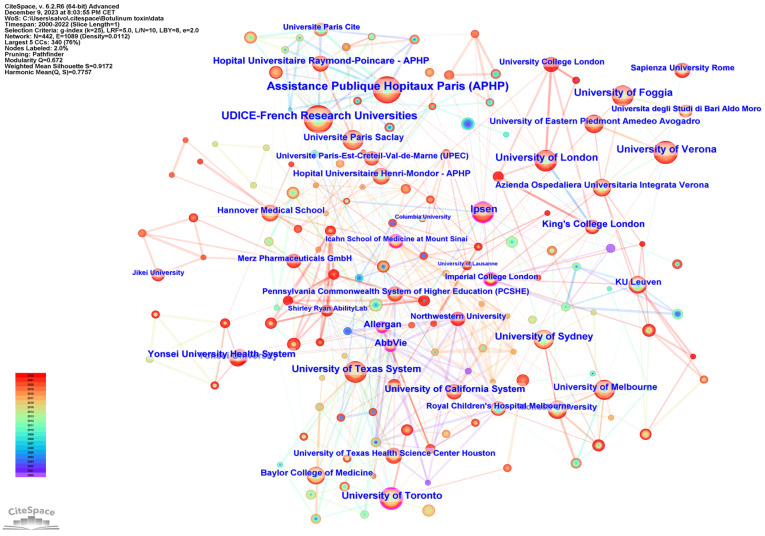
Network cooperation map of institutions. The nodes represent institutions, and the lines between the nodes represent cooperative relationships. The larger the node size, the larger the number of publications. The nodes in the outermost area with purple rings indicate high centrality.

Figure 4 shows the network cooperation map of institutions; each node represents a different institution, and the larger the node, the higher the output of the institution. The color of the rings indicates the year of publication. The highest centrality was found at Imperial College (0.17), followed by Icahn School of Medicine at Mount Sinai (0.14), and the University of Toronto (0.13). A purple outer ring is present when the betweenness centrality is greater than 0.1, and the node is considered critical.

### 2.5. Journals Analysis

A total of 394 journals published articles on the use of BoNT-A in spasticity. Table 3 presents the top ten journals and co-cited journals that published articles on the use of BoNT-A in spasticity. The journal with the largest number of publications was *Toxins* (81 publications, 5.44%), followed by the *Journal of Rehabilitation Medicine* (67 publications, 4.50%), *Archives of Physical Medicine and Rehabilitation* (48 publications, 3.22%), and *Developmental Medicine and Child Neurology* (44 publications, 2.95%). The highest-ranking journal was the *European Journal of Physical and Rehabilitation Medicine*, with an impact factor of 5.313, followed by *Toxins* with an impact factor of 5.075; these were the only two journals in the top 10 with an impact factor greater than 5.000. Five journals had an IF between 3.000 and 5.000, while three journals had an IF < 3.000 (minimum 2.218).

*Archives of Physical Medicine and Rehabilitation* was the most co-cited journal, with 964 co-citations, followed by *Neurology* (870 co-citations), the *European Journal of Neurology* (840 co-citations), *Clinical Rehabilitation* (729 co-citations), and the *Journal of Neurology, Neurosurgery, and Psychiatry* (701 co-citations). Among the top 10 co-cited journals, six journals had impact factors higher than 4.500, and two journals had impact factors higher than 12.000.

### 2.6. Authors Analysis

**Figure 5:**
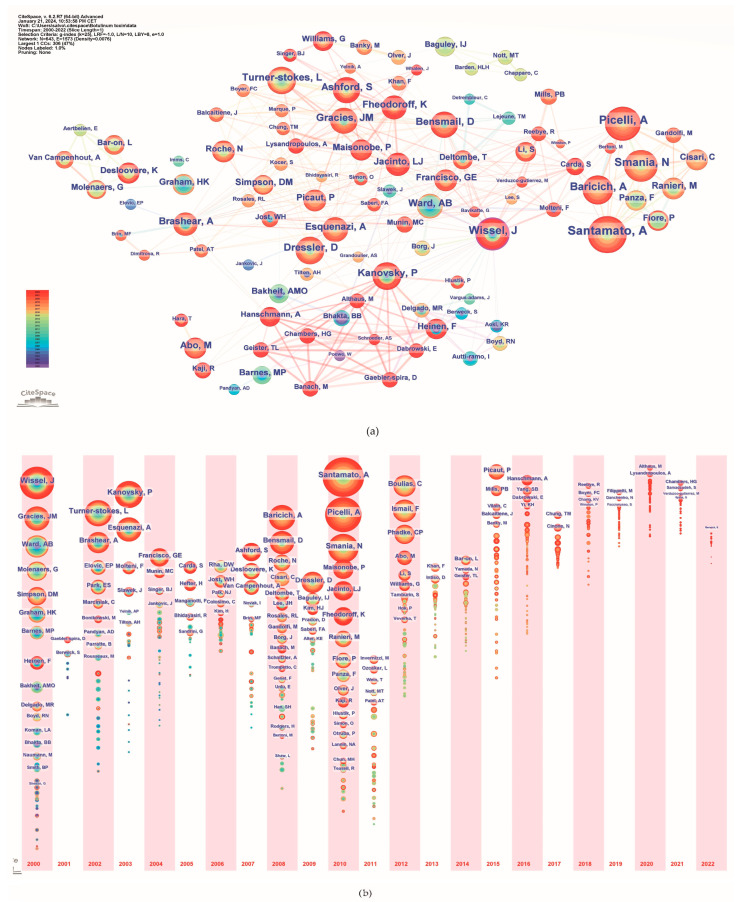
Author analysis. (**a**) Collaboration networks; (**b**) Timeline. The colors of the circles represent the years from 2000 to 2022.

A total of 5039 authors published papers on the use of BoNT-A in spasticity between 2000 and 2022. Table 4 lists the top 15 most active authors and their related information. They published 306 papers, accounting for 20.56% of the total number of papers. The top three ranked authors by publication count were Andrea Santamato from Italy, Alessandro Picelli from Italy, and Jörg Wissel from Germany. Figure 5a illustrates the network of interconnected authors contributing to spasticity research, identifying key individuals based on the volume of their publications. The nodes, differentiated by color and size, represent authors, with size denoting the number of publications and color indicating the year of publication. A timeline view analysis of the authors is shown in Figure 5b. The authors that exhibited a high degree of centrality include Wissel J., with a centrality score of 0.12; Picelli A., with a centrality score of 0.07; Turner-Stokes L., Kanovsky P., Ward A., Molteni F., and Novak I., each with a centrality score of 0.05; and Brashear A. and Bensmail D., both with a centrality score of 0.04.

**Figure 6:**
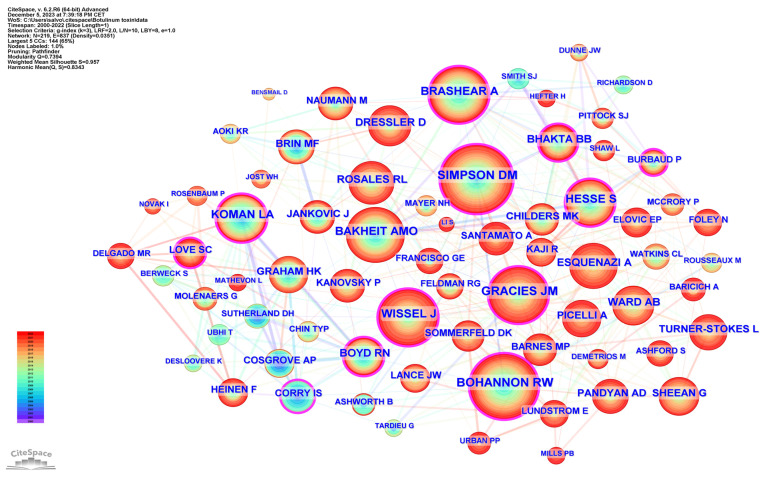
Co-cited authors.

Table 5 lists the top 15 co-cited authors and their related information. The top three ranked authors by publication count were Simpson, David M. from the USA, Bohannon, Richard W. from the USA, and Gracies, Jean Michel from France. Figure 6 delineates the network of prolific authors whose work has garnered significant citations and maps the intellectual structure and collaborative patterns among influential authors in spasticity research.

### 2.7. Analysis of References

A total of 24,823 references were cited in publications relating to the use of BoNT-A in spasticity treatment during the 20 years between 2000 and 2022. The 1489 publications were cited 39,338 times (26,708 times after removing self-citation). We present a detailed citation analysis of the most influential studies on this topic. To elucidate temporal trends in the research landscape, we stratified the most cited references into two distinct temporal blocks. The first block encompasses the period from 2000 to 2011, highlighting seminal works and formative ideas that provided a foundation for subsequent developments in the field. The second block, from 2012 to 2022, captures the most current and impactful research, reflecting the evolution of thought and latest scientific advancements. The top 10 references with the most citations are presented in Table 6 and Table 7.

**Figure 7:**
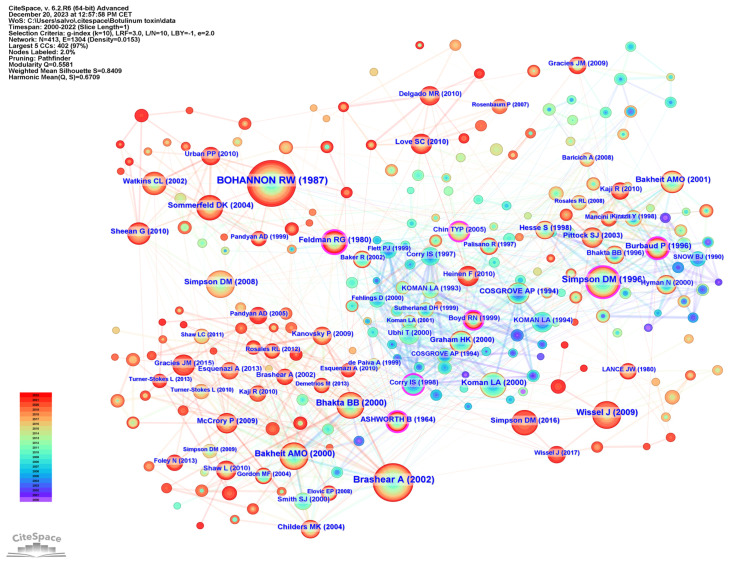
Reference co-citation analysis.

To examine publication citations, a co-citation analysis was conducted on the cited references. This analysis involved 413 co-cited references interconnected through 1304 links, as depicted in Figure 7. The most frequently co-cited (360 co-citations) was “Interrater reliability of a modified Ashworth scale of muscle spasticity” published by Bohannon et al. in *Physical Therapy* in 1987, followed by “Intramuscular Injection of Botulinum Toxin for the Treatment of Wrist and Finger Spasticity after a Stroke” (249 co-citations) by Brashear et al. in *New England Journal of Medicine* in 2002, and “Botulinum toxin type A in the treatment of upper extremity spasticity: a randomized, double-blind, placebo-controlled trial” (191 co-citations) by Simpson et al. in *Neurology* in 1996.

**Figure 8:**
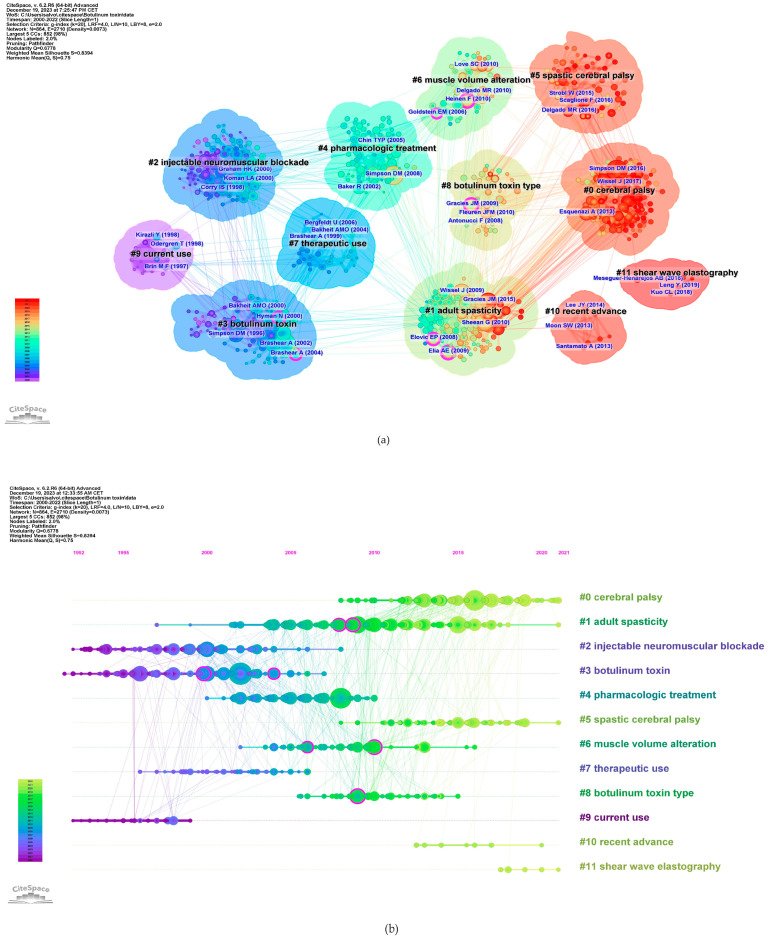
Reference co-citation analysis. (**a**) Clustered network of co-cited references from publications (12 clusters). Shades of color indicate different times of publication, with warmer colors indicating newer publications. The purple nodes represent literature with centrality. (**b**) Timeline map. In the figure, the Cluster numbers are sequentially arranged from #0 to #11.

The co-citation co-occurrence cluster map (Figure 8a) reveals 12 clusters with a q-value of 0.75 and a silhouette value greater than 0.8. In this analysis, each cluster represents a collection of studies or references that frequently cite each other or share common keywords, indicating a specific topic or theme within the broader research domain. The size of each cluster may reflect the volume of research, and the intensity of the connections between nodes within a cluster can denote the strength of the relationships or the centrality of certain references within that topic. The largest clusters were #0 CP, #1 adult spasticity, and #2 injectable neuromuscular. Figure 8b presents a timeline visualization, mapping the chronological development of the research clusters over time. Each horizontal line corresponds to a cluster, with individual studies represented as nodes whose size reflects citation impact. The color gradient from cool to warm hues represents the progression of time, with newer research represented by warmer colors. This timeline shows the emergence, growth, and current state of research topics within the field, highlighting the shifts in focus and attention over years.

**Figure 9:**
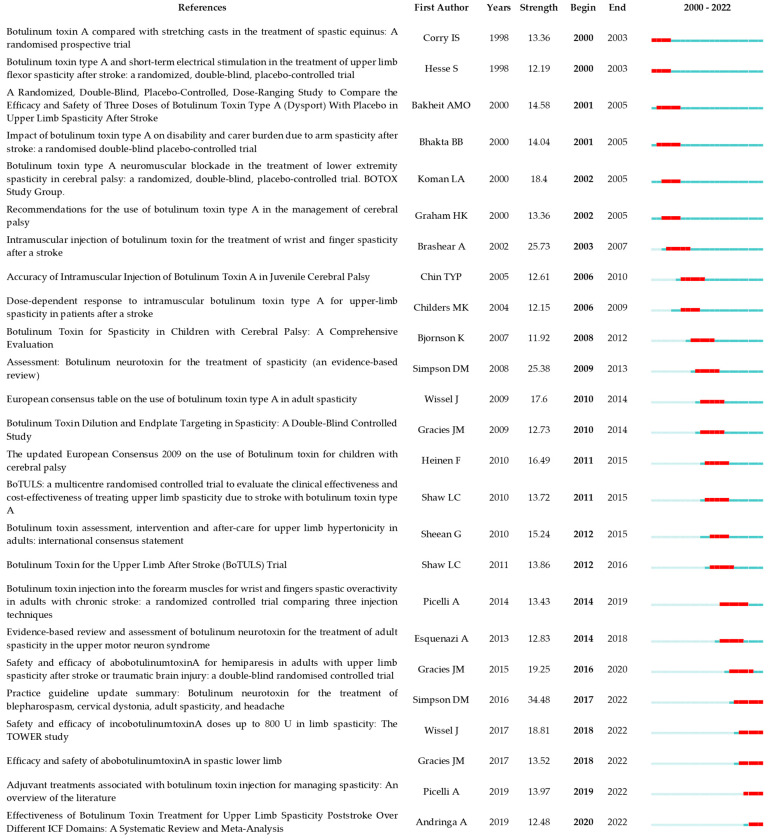
The top 25 references with the strongest citation bursts. The minimum duration of the burst was set at 2 years. γ was set at 1.0. The red segment on the timeline denotes the outbreak era and the blue line represents the timeframe. References list: [12,15,17,21,24,26,27,29,32,38,39,40,41,42,43,44,45,46,47,48,49,50,51,52,53].

The top 25 co-cited references with the strongest citation burst can be observed in Figure 9. Of these, the article with the strongest burst is “Practice guideline update summary: Botulinum neurotoxin for the treatment of blepharospasm, cervical dystonia, adult spasticity and headache”, published in *Neurology* by Simpson et al. in 2016. “Intramuscular injection of botulinum toxin for the treatment of wrist and finger spasticity after a stroke”, published in *New England Journal of Medicine* by Brashear et al. in 2002 is the second strongest, and the third is “Assessment: Botulinum neurotoxin for the treatment of spasticity (an evidence-based review)”, published in *Neurology* by Simpson et al. in 2008.

### 2.8. Keywords Analysis

Keywords may reflect current topics and present the frontiers of research that are garnering heightened interest.

**Figure 10:**
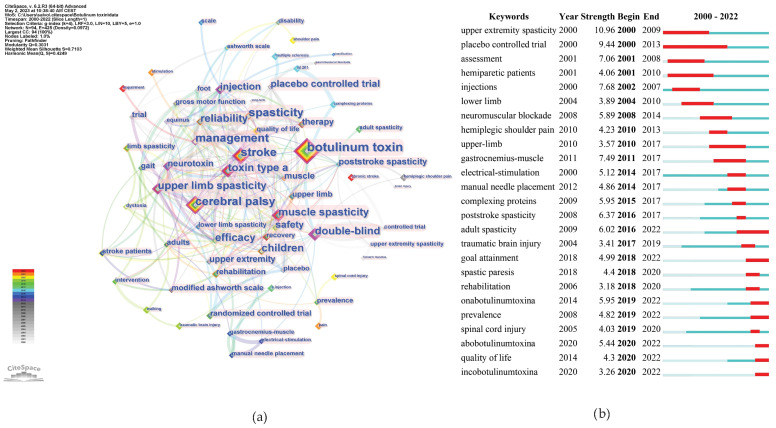
Keywords analysis. (**a**) Co-occurrence counts; (**b**) bursts.

As shown in Figure 10a, the top three keywords with the highest occurrence were botulinum toxin, stroke, and CP.

Figure 10b shows the most recent burst keywords, i.e., the current frontiers that may require further effort to enhance related research. We selectively included keywords demonstrating a significant surge in academic relevance, as indicated by a keyword burst, in the year 2022. This criterion ensures the inclusion of keywords that not only hold contemporary significance, but are also indicative of emergent trends and foci within the current scientific narrative. Among these keywords, adults’ spasticity, OnabotulinumtoxinA, AbobotulinumtoxinA, and goal attainment had the strongest burst strength.

The top 150 keywords, with associated metrics, are listed in Supplementary Table S1.

**Figure 11:**
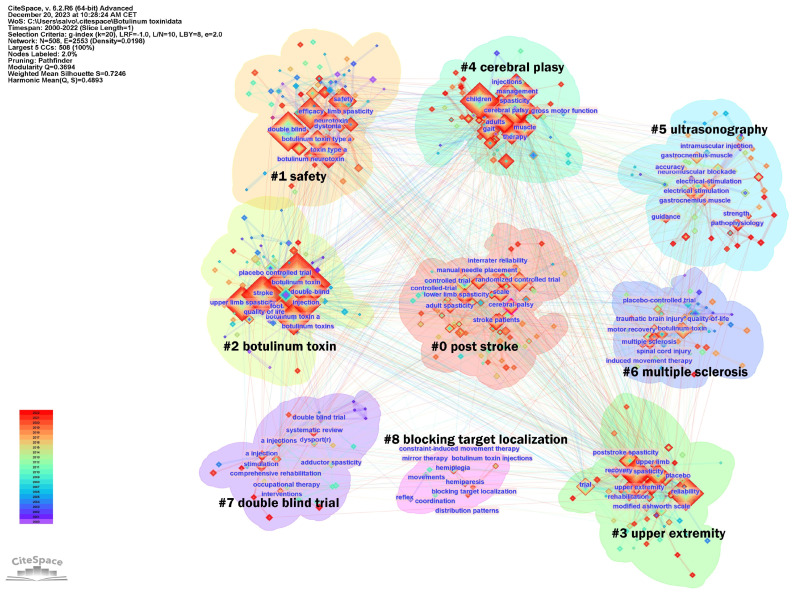
Keywords analysis clusters. In the figure, the Cluster numbers are sequentially arranged from #0 to #11.

In addition, the keywords were divided into nine clusters, as shown in Figure 11. The size of the nodes reflects the prevalence of keywords in the literature, and the connecting lines suggest thematic links. This map provides an overview of the diverse and developing research domains concerning botulinum toxin application in neurology.

The largest cluster identified was #0 *post-stroke*, suggesting a significant volume of research dedicated to post-stroke spasticity. Clusters #0 and #4 focus on specific neurological conditions, such as CP and stroke. Cluster #1 *safety* and #2 *botulinum toxin* address the toxin’s safety and efficacy, indicating concentrated studies on safety protocols and risk assessment in treatments. Cluster #3 *upper extremity* points to research concentrated on spasticity management in the arms, possibly exploring functional outcomes. Cluster #5 *ultrasonographic* investigates the precision of toxin injections using ultrasonographic guidance and the importance of skeletal muscle evaluation, indicating a trend towards incorporating technology for improved accuracy and assessment. Cluster #6 *multiple sclerosis* exhibits interest in the management of spasticity in non-stroke populations, including patients with multiple sclerosis, traumatic brain injury, or spinal cord injury. Finally, the presence of clusters such as #7 *double blind trial* and #8 *blocking target localization* indicate a commitment to rigorous clinical trial designs and the exploration of anatomically precise treatment modalities, respectively, underscoring a dynamic and methodical research landscape aiming to refine therapeutic strategies for spasticity.

## 3. Discussion

### 3.1. Research Status

The bibliometric analysis conducted on research examining the application of BoNT-A to spasticity from 2000 to 2022 reveals a compelling picture of scientific advancement and collaboration. The annual trend of publications displays a significant growth trajectory, with a noteworthy increase in research output starting in 2007 and peaking in 2020, which may reflect the heightened recognition of BONT-A’s potential in spasticity treatment following pivotal studies.

Country-level contributions highlight the United States, United Kingdom, Italy, Germany, and France as leading forces in this domain, with the United States producing a substantial volume of research. Institutional analysis illuminates the significant roles played by key establishments such as Assistance Publique Hopitaux Paris and UDICE-French Research Universities. The centrality of these institutions in the network cooperation map underscores their influence and the importance of collaborative ecosystems for driving research frontiers.

Research on BoNT-A in spasticity is well-distributed across 394 journals, indicating wide interest across various medical disciplines. Leading the publication count is *Toxins*, with *Journal of Rehabilitation Medicine* and *Archives of Physical Medicine and Rehabilitation* closely following, showcasing their prominence in this research area. *Toxins* and *the European Journal of Physical and Rehabilitation Medicine* stand out as having the highest impact factors, reflecting the significant influence of the research they publish. Co-citation data point to *Archives of Physical Medicine and Rehabilitation* as the most referenced journal.

The field of BoNT-A research on spasticity over the past two decades has been profoundly influenced by a network of dedicated researchers, whose collaborative efforts have advanced both the scientific and clinical understanding of its use. This network of researchers is led by Andrea Santamato, with 41 publications since 2010, marking a significant contribution across clinical trials and therapeutic explorations. Following closely, Alessandro Picelli’s 36 publications have been influential in defining treatment protocols. Jörg Wissel, with a research history starting in 2000 and encompassing 34 publications, has played a key role in the early and evolving clinical application of botulinum toxin. The size of nodes on the bibliometric map and their citation counts reflects the resonance their work has had within the scientific community (Figure 5). The bibliometric timeline further reveals other important details of authors whose contributions have shaped the field of botulinum toxin use in spasticity. In the early 2000s or before, authors such as Jörg Wissel, Anthony Ward, and Jean Michel Gracies began to solidify their positions as influential figures in the field. Their early research set the stage for the evolution of botulinum toxin applications in spasticity, contributing to the understanding of the pathophysiology of spasticity and influencing treatment paradigms and research directions. Midway through the first decade of the 2000s, we noted the emergence of Lynne Turner-Stokes and Allison Brashear, whose research outputs expanded the field, particularly in relation to the stroke population. Furthermore, the contributions represented by Alberto Esquenazi and Peter Kanovsky’s clinical research have often translated into practice-changing protocols for the management of spasticity. Entering the 2010s, the influence of Italian authors such as Andrea Santamato, Alessandro Picelli, Nicola Smania, and Alessio Baricich became prominent. Other authors, such as Djamel Bensmail, Farooq Ismail, and Chris Boulias also demonstrated substantial contributions.

Global influence in the field is further underlined by authors like David M. Simpson and Richard W. Bohannon from the USA, Abdel Magid Bakheit, Anthony Ward, and Bipinchandra Bhakta from the United Kingdom, and Stefan Hesse and Dirk Dressler from Germany, whose high co-citations and high h-index numbers underscore their authority and impact in the field. Collectively, these authors have not only expanded the scope of botulinum toxin use in spasticity, but have also refined associated therapeutic approaches, ensuring that treatment is both effective and tailored to the specific requirements of each patient.

### 3.2. Research Hotspots and Trends

A research hotspot is a specific area where there is a concentrated and intense focus on research activity at a given time, whereas a research trend is a broader, longer-term movement in the field of study. Such trends and research hotspots can be discerned through keyword co-occurrence and cluster analysis [19]. Analysis of co-occurring keywords used in articles on the use of BoNT-A in spasticity published between 2000 and 2022 yielded nine clusters: *post-stroke, safety, botulinum toxin, upper extremity, cerebral palsy, ultrasonography, multiple sclerosis, double-blind trial,* and *blocking target localization* (Figure 11). The provided cluster analysis unveils a multifaceted research domain, where studies span from the critical role of BoNT-A in the management of spasticity across various conditions and patient populations, to the safety profile and precise application of BoNT-A.

The *botulinum toxin* cluster illustrates concentrated investigation into the toxin’s efficacy and optimization for spasticity treatment. The research encapsulated within this cluster demonstrates the mechanism of action of BoNT-A and its therapeutic applications. This cluster intersects notably with the *post-stroke* and *upper extremity* clusters, highlighting the role of the toxin in addressing post-stroke muscle spasticity, particularly in the upper limbs. The interweaving of these themes shows a holistic approach to post-stroke care, where BoNT-A serves as a critical element in alleviating the debilitating effects of spasticity and enhancing patient quality of life. Concurrently, there is a dedicated stream of research on CP, emerging in the specific cluster *cerebral palsy*, which navigates through the complexities of treating this condition in both children and adults, pointing to a lifecycle approach in therapy. The *multiple sclerosis* cluster expands the spasticity research to include adult non-stroke patients, focusing on the specific challenges and treatments for managing spasticity in these populations.

The *ultrasonography* cluster encapsulates research dedicated to the use of ultrasound technology in the context of neurological and muscular disorders, particularly in the evaluation and treatment of spasticity. Within this cluster, the primary focus is on how ultrasonography can be employed not only as a means of guiding interventions, such as botulinum toxin injections, but also as a diagnostic tool to visualize muscles and surrounding tissues. This cluster discusses the advantages that ultrasonography provides for medical treatments via enhancing the accuracy of injections, allowing clinicians to identify optimal locations for administration. Additionally, the cluster includes research on the role of ultrasonography in evaluating post-treatment muscle changes, such as alterations in muscle thickness and fibrosis, which could be critical in assessing the efficacy of spasticity management strategies. The incorporation of ultrasonography into treatment protocols represents a significant advancement in personalized medicine, allowing tailored interventions based on real-time anatomical and functional data. The presence of *double-blind trial* in the clusters reaffirms the commitment to methodological rigor amongst the research community, ensuring that findings in comprehensive rehabilitation and stimulation therapies stand up to the highest scrutiny. Finally, the focus on *blocking target localization* indicates a trend towards precise strategies to optimize the therapeutic efficacy. Collectively, these clusters reveal a landscape in which the confluence of safety, efficacy, and precision shapes the contours of current research in the treatment of spasticity.

In bibliometric analysis, the evolution of research themes over time is often tracked through the frequency and patterns of keywords in the scientific literature. Such an analysis investigates where the academic community has focused its collective efforts over time and acts as an indicator of the changing landscape of academic interest. Examining the evolution of keywords, it is evident that over the past few decades, research on the application of botulinum toxin for spasticity has changed.

In the early 2000s, the research landscape was primarily anchored in laying the foundational groundwork, with a strong focus on *upper extremity spasticity* and *placebo-controlled trials*. This era was crucial in establishing the baseline efficacy of treatments, particularly emphasizing upper-limb spasticity. Concurrently, there was an emerging interest in the assessment and understanding of the specific responses of *hemiparetic patients*, indicating an early shift towards patient-specific outcomes and comprehensive treatment evaluation.

As the mid-2000s approached, the focus broadened, encapsulating *lower limb* spasticity and delving into the mechanisms of *neuromuscular blockade*. This period marked a significant expansion in the scope of research, moving beyond the upper limbs to a more holistic understanding of the management of spasticity. The field began to mature with emerging approaches, particularly in addressing the specific challenges of *hemiplegic shoulder pain*.

The 2010s represented an era of integration and innovation, characterized by the exploration of strategies such as *electrical stimulation* and *manual needle placement* as critical tools for guiding the injection of toxins, underscoring the importance of accuracy and precision in administering treatments. Research also focused on specific muscles, as evidenced by the interest in the *gastrocnemius muscle*, which plays a key role in equinovarus, a foot deformity commonly observed in conditions such as stroke, CP, and traumatic brain injuries. Concurrently, there was a pronounced emphasis on safety and long-term efficacy, particularly in the study of *complexing proteins*. Some evidence has shown that complexing proteins do not influence the stability of BoNT-A in its finished formulated drug products, and their effect on neurotoxin diffusion appears negligible [54]. However, the potential contribution of these proteins to immunogenicity, and thereby to the risk of treatment failure due to antibody-induced therapy resistance, remains an area of active research [55]. Recent studies have suggested that the clinical significance of complexing proteins is in their induction of immunogenic responses; however, this requires further investigation [56].

Entering the late 2010s and the early 2020s, the narrative took a decisive turn towards personalized management and diversification of treatment options. The emphasis shifted to *goal attainment*, *quality of life,* and *rehabilitation*, reflecting a profound commitment to individualized outcome-based treatment plans. This period also witnessed a significant surge in citations for specific botulinum toxin types, such as *OnabotulinumtoxinA*, *AbobotulinumtoxinA*, and *IncobotulinumtoxinA,* unveiling a growing interest in the distinct properties and efficacies of various formulations. This latest phase encapsulates a holistic approach, in which treatments are increasingly tailored to individual needs, with a heightened focus on enhancing the overall quality of life of patients.

Throughout these evolving phases, the field has not only grown in depth of understanding and complexity but has also demonstrated responsiveness to emerging challenges and opportunities. It reflects a dynamic journey from establishing fundamental knowledge and assessing treatment efficacy to embracing a multifaceted, patient-centered approach in recent years, signaling a future where therapy is increasingly personalized and aligned with the needs of patients.

### 3.3. Research Frontiers and Knowledge Bases

Research frontiers are the most advanced areas in the field of study, where scientists explore new and uncharted topics. These are the areas where the latest and most innovative research has occurred, often leading to discoveries and breakthroughs. A research frontier is a cluster of articles actively cited by researchers [19]. In our bibliometric analysis using CiteSpace, we identified a series of prominent research frontiers that represent the collective knowledge base within the field of spasticity and BoNT-A applications. Our analysis yielded 12 distinct clusters, each named using key terms derived from the content of the articles within, signifying the focal points of research efforts. To articulate the core literature that constitutes the foundation of each research frontier, we summarized the most frequently cited and influential articles in each cluster (Figure 8). These crucial articles serve as the pillars of their respective research frontiers in the domain of spasticity management and therapeutic use of botulinum toxin. In summary, we have identified six research frontiers.

#### 3.3.1. Early Adoption and Applications of Botulinum Toxin in Spasticity

Botulinum toxins are produced by various Clostridium species and are composed of two peptide chains linked by a disulfide bond, with significant variations in their amino acid sequences among different serotypes and subtypes [57]. The molecular structure of BoNT-A is characterized by three distinct domains: the heavy chain, which specifically binds to neurons, facilitating the toxin’s entry; the translocation domain, responsible for translocating the light chain into the neuronal cell cytosol; and the enzymatically active light chain, which cleaves specific SNARE proteins, disrupting neurotransmitter release by blocking vesicle fusion on the inner surface of cellular membrane [58]. This structure allows BoNT-A to effectively inhibit acetylcholine release at neuromuscular junctions, leading to reversible muscle paralysis [57]. The duration of BoNT-A’s action varies, typically lasting several months, as the neuron gradually recovers function either through the sprouting of new synaptic contacts or the regeneration of cleaved SNARE proteins, thereby restoring neurotransmission.

BoNT-A has been developed into three distinct injectable formulations for clinical applications in spasticity: OnabotulinumtoxinA (Botox), AbobotulinumtoxinA (Dysport), and IncobotulinumtoxinA (Xeomin) [59]. Apart from spasticity indications, there are various other formulations available, including DaxibotulinumtoxinA, LetibotulinumtoxinA, and PrabotulinumtoxinA [57]. Regarding spasticity treatment, all three BoNT-A formulations have received Food and Drugs Administration (FDA) approval [60]. OnabotulinumtoxinA, first approved by the FDA in 1989 for strabismus and blepharospasm, gained its inaugural approval for spasticity management on 10 March 2010, specifically for the treatment of upper limb spasticity in adults; this approval was expanded on 29 July 2021. AbobotulinumtoxinA initially received FDA approval on 30 April 2009 for cervical dystonia and glabellar lines and obtained approval for upper limb spasticity on 17 July 2015. IncobotulinumtoxinA was approved for cervical dystonia and blepharospasm on 2 August 2010, and was the first treatment approved for adult upper limb spasticity on 23 December 2015. Notably, the regulatory standards for the use of botulinum toxin for spasticity vary among different countries. The three formulations of BoNT-A, which share the same fundamental mechanism of action, exhibit variations in the quantity of neurotoxins, complexing protein sizes, excipient composition [61], and potency [62]. Furthermore, other differences, such as dilution and the potential for inducing neutralizing antibodies may further differentiate their clinical profiles (i.e., efficacy, duration of effect, and adverse events) [59]. The comprehensive impact of these dissimilarities on clinical outcomes remains an area of ongoing investigation and has not yet been fully elucidated. Owing to these distinctions, it is essential to acknowledge that these formulations are not interchangeable in clinical practice [63]. Each formulation requires specific consideration in terms of dosage, administration, and expected outcomes.

#### 3.3.2. The Role of Botulinum Toxin in Pediatric Populations

The use of BoNT-A has become increasingly prevalent in the management of childhood spasticity, particularly CP. The primary indication for BoNT-A therapy in CP is focal muscle overactivity, which is key to improving gait and function in ambulatory children. Additionally, BoNT-A is used in the upper limbs to enhance posture and function.

Despite initial uncertainties due to the lack of approved treatment protocols, BoNT-A has shown significant promise in controlling excessive muscle contraction in specifically targeted muscles. Koman et al.’s 2004 review in The Lancet outlined a comprehensive approach to managing CP, highlighting advancements since the 1980s in treatments, ranging from physical therapy to surgery [22]. The review particularly noted the efficacy of BoNT-A injections, which, when integrated with physiotherapy and orthotic support, can significantly improve motor function, minimize the need for oral spasticity medications, and when combined with physiotherapy and orthotics, enhance overall treatment efficacy [22]. Initiating treatment with BoNT-A early, preferably when the patient is between 1 and 5 years old, is essential for optimizing its therapeutic benefits, which may include reducing the necessity for treating contractures and postponing surgeries [15]. However, in older children, the presence of fixed contractures can limit BoNT-A effectiveness [15].

“The updated European Consensus 2009 on the use of botulinum toxin for children with cerebral palsy” by Heinen et al. presented a comprehensive framework for best practices in using BoNT-A for treating children with CP, integrating clinical experiences from 36 European treatment centers [17]. CP is the most common cause of spastic movement disorders in children. This consensus recognizes the importance of BoNT-A in improving the overall management of CP, particularly in enhancing the functional abilities and quality of life of affected children. Moreover, it emphasizes a paradigm shift from viewing BoNT-A as a standalone treatment to considering it a supportive therapy among a range of conservative and surgical strategies, representing an interdisciplinary, multimodal team approach [17]. In 2010, Love et al. compiled an international consensus statement focusing on the assessment, intervention, and after-care of lower limb spasticity in children with CP, particularly addressing the use of BoNT-A [64]. This paper represents an expert review that synthesized data from various randomized clinical trials and offers comprehensive treatment recommendations, highlighting the importance of a multidisciplinary approach in assessing and evaluating the outcomes of BoNT-A injections in this patient population. In the same year, Delgado et al. conducted a comprehensive review of the efficacy and safety of pharmacological treatments for childhood spasticity caused by CP [65]. They systematically reviewed the literature from 1966 to July 2008 and found that for localized/segmental spasticity, BoNT-A is effective in reducing spasticity in both the upper and lower extremities, although there is conflicting evidence regarding its impact on functional improvement. BoNT-A has generally been considered safe for use in children with CP, with only occasional reports of generalized weakness. Additionally, its application at high doses in both children and young adults has proven safe, with minimal adverse events (rash, urinary incontinence, and mild generalized botulism) observed [66]. Furthermore, its long-term administration has shown an acceptable safety profile, marked by a slightly higher rate of adverse events in treated individuals (approximately 25%) than in control groups (15%), demonstrating its tolerability over repeated injections [67]. The incidence of serious adverse events following BoNT-A injections in 1147 children out of 1980 injection episodes was found to be low, with 1% experiencing incontinence and 1.3% resulting in unplanned hospital admissions due to respiratory symptoms. These adverse events were more likely to be related to higher Gross Motor Function Classification System (GMFCS) levels and larger BoNT-A doses [68]. Although BoNT-A is generally regarded as safe and suitable for managing localized spasticity, clinicians should be cautious, particularly given concerns about potential distant spread. It is vital to identify specific treatment goals and ensure close monitoring after injections to balance any potential risks with benefits to the child [69].

A 2013 review by Novak et al. systematically evaluated the efficacy of various treatments and interventions for children with CP, highlighting BoNT-A as an effective intervention for managing spasticity in CP [28]. This paper is noteworthy as it is presently the most cited work in this area, with 757 citations, underscoring its influence and importance.

In 2016, Strobl et al. emphasized the importance of individualized assessment, setting specific treatment goals, and integrating BoNT-A with other therapies in children with CP [70]. Their findings support the safety and effectiveness of BoNT-A, particularly for children with GMFCS levels I to III, while recommending a multimodal treatment approach and cautious dosage management to optimize motor development and function.

M. R. Delgado advanced our understanding of CP and associated treatment approaches through a series of impactful studies [71,72]. Initially focusing on lower limb spasticity, his research team first established the efficacy of AbobotulinumtoxinA in treating localized spasticity in 2010, with subsequent studies in 2016 and 2018 confirming its effectiveness and safety for conditions such as dynamic equinus foot deformity [65,73,74]. These studies highlighted improvements in muscle tone, gait, and functional goals, and demonstrated the sustained benefits of BoNT-A across multiple treatment cycles. Shifting focus to upper limb spasticity, a 2020 study expanded the applications of BoNT-A by demonstrating its efficacy in reducing upper limb spasticity [75], and in 2022 further explored this by analyzing dosing and muscle injection patterns [76].

OnabotulinumtoxinA’s journey in CP began with early trials in the 1990s, showing efficacy in reducing spasticity, and progressed through critical double-blind trials and comprehensive Phase 2 studies, affirming its safety and effectiveness in improving gait function [40,77]. Recent studies have reinforced the efficacy and safety of OnabotulinumtoxinA in treating both upper and lower limb spasticity in CP, after its first FDA approval in 2019 [78,79]. In parallel, the therapeutic potential of IncobotulinumtoxinA has been explored in a series of determining studies: the TIM (Treatment with IncobotulinumtoxinA in Movement) study [80], the XARA (incobotulinumtoXinA in aRm treatment in CP) [81] study, and the TIMO (Treatment with IncobotulinumtoxinA in Movement Open-Label) study [82]. Recent systematic reviews by Farag et al. and Klein et al. have focused on upper limb spasticity in children with CP [83,84], whereas a Cochrane review by Blumetti et al. addressed treatments for lower limb spasticity in this population [85]. Finally, recent studies have highlighted the need for a careful balance between the benefits and potential harm of BoNT-A, especially in regard to long-term use. Concerns have been raised regarding muscle atrophy, sustained reductions in muscle strength, and a loss of contractile elements associated with BoNT-A use [86,87,88]. These effects may not result in long-term functional improvement. This further underscores the importance of employing BoNT-A treatment within a multimodal approach that not only considers the weakening of specific muscles to alleviate spasticity but also incorporates strategies for strengthening other muscles to maintain or enhance overall function.

#### 3.3.3. The Role of Botulinum Toxin in Adult Populations

The application of botulinum toxin in adult spasticity includes its use in stroke and non-stroke patients, highlighting its role in improving functional outcomes and quality of life.

We analyzed the role of BoNT-A in managing spasticity in patients with stroke, drawing on a range of influential studies in the field. Spasticity occurs in 20–30% of all stroke patients, more commonly in the upper limbs than in the lower limbs, and seems to be more prevalent among younger patients [33]. Initially, studies such as those by Sommerfeld et al. and Bhakta et al. laid the foundation for understanding the prevalence and impact of post-stroke spasticity. Sommerfeld et al. highlighted that while spasticity contributes to motor impairments and activity limitations, it was present in only 19% of the stroke patients included at 3 months post-stroke, suggesting a need for careful evaluation before deciding on rehabilitation approaches [20]. Bhakta et al. demonstrated the effectiveness of BoNT-A in reducing disability and carer burden in patients with chronic stroke and upper limb spasticity, albeit observing the potential for muscle weakness following treatment [89]. Subsequently, Brashear et al. conducted a placebo-controlled trial showing that BoNT-A significantly improved flexor tone in the wrist and fingers post-stroke, with patients reporting greater improvement in selected areas of disability such as hygiene and dressing [21]. Elovic et al. assessed the safety and effects of repeated treatments with BoNT-A on functional disability, quality of life (QOL), and muscle tone in patients with upper limb post-stroke spasticity. They highlighted that repeated treatments with BoNT-A every 12 weeks for up to 56 weeks were well tolerated and significantly improved outcomes [90].

To understand the broader implications of BoNT-A treatment, the multicentric “Botulinum Toxin for the Upper Limb after Stroke” (BoTULS) study was undertaken. While this study did not find a significant enhancement in overall upper limb function following BoNT-A treatment, it did observe improvements in muscle tone, strength, and performance in specific functional tasks [45]. This was further explored by Shaw et al., who concluded that BoNT-A might not significantly improve active upper limb function, but could benefit basic tasks and pain management [47].

Elia et al. [91], Wissel et al. [12], and Esquenazi et al. [49] provided a broader perspective on upper and lower limb spasticity treatment. Wissel et al. emphasized the value of BoNT-A in managing spasticity following acquired brain injury, including stroke, and advocated further studies addressing active function. Elia et al. and Esquenazi et al. reinforced the efficacy of BoNT-A in reducing muscle tone and improving passive function, while also noting less robust improvements in active function [49,91]. Both studies revealed fewer studies for lower limb spasticity and the need for further good-quality studies assessing the efficacy of BoNT-A on lower limb spasticity. A paper with a recent citation burst, “Efficacy and safety of AbobotulinumtoxinA in spastic lower limb” by Gracies et al. [92] (Figure 9), found that after a single injection of BoNT-A, there were significant improvements observed in muscle tone in the gastrocnemius–soleus complex, and that these improvements continued with repeated treatments. Importantly, this study reported an increase in comfortable barefoot walking speed and a greater likelihood of achieving community ambulation over the course of the year.

In conclusion, while the majority of existing studies have concentrated on the upper limbs, the growing focus on lower limb treatment represents an important evolution in the field. As research continues to evolve, it is anticipated that treatments will increasingly address the full spectrum of spasticity-related challenges faced by patients to avoid complications, thereby enhancing the effectiveness of rehabilitation strategies in post-stroke care and improving patient quality of life [93]. Furthermore, current trends suggest a pivot towards optimizing BoNT-A’s use on its own or in conjunction with other therapies to maximize patient outcomes, as we will analyze in the next paragraphs.

In addition to stroke, botulinum toxin has demonstrated significant benefits in various neurological conditions in the adult population. Spasticity is a crucial early consequence of severe brain injury, often leading to lower limb deformities and hindering successful rehabilitation. In patients with traumatic brain injury, botulinum toxin has been shown to substantially improve spasticity and range of motion, particularly in the upper extremity [94]. This improvement aids in the performance of daily activities and enhances the quality of life of these patients. Verplancke et al. conducted a randomized controlled trial to determine whether serial casting combined with botulinum toxin could reduce the development of calf contracture after severe head injury [95]. The results indicated that casting with botulinum toxin was safe in maintaining a positive passive range of ankle motion; however, further investigations are needed to confirm its effectiveness.

In multiple sclerosis, spasticity is a common complication, affecting approximately 80% of patients, predominantly in the lower limbs. BoNT-A injections have been shown to provide pain relief and, at higher doses, lead to a notable reduction in spasticity in these patients, thus facilitating easier care and rehabilitation [96]. Hyman et al. underscored the efficacy of BoNT-A in reducing hip adductor spasticity in multiple sclerosis, demonstrating its benefits despite the concurrent use of oral anti-spasticity medication and analgesics [97]. The optimal dose for managing hip adductor spasticity was suggested to be between 500 and 1000 units, divided between both legs, indicating a dose-dependent improvement in spasticity and pain management in multiple sclerosis patients [97].

Spasticity affects 65% to 78% of individuals with spinal cord injury (SCI), particularly those with cervical and higher-thoracic-level injuries. It often hinders function, ambulation, positioning, and hygiene, and may cause pain [98]. The use of BoNT-A for managing spasticity in patients with SCI has been explored with promising results, although the number of studies conducted with patients with SCI is relatively small. This lack of high-quality evidence emphasizes the need for further research to validate BoNT-A’s effectiveness and safety in these individuals [99]. Yan et al. found that BoNT-A effectively managed spasticity in spinal cord injury, demonstrating notable improvements in muscle tone and functional activities. The study, which compared BoNT-a with baclofen and physical therapies, highlighted its distinct advantages and safety profile for treating such conditions. Moreover, another study reported significant improvements in muscle tone, goniometric performance, and pain relief in patients with focal spasticity treated with BoNT-A. Early treatment (within the first six months of SCI) led to greater improvements, with a safety profile characterized by minimal adverse reactions. Interestingly, patients with incomplete injuries and specifically those with ASIA D injuries showed more pronounced improvements [100]. In patients with hereditary spastic paraplegias, BoNT-A was found to be effective in reducing spasticity and improving gait quality without significantly altering muscle strength [101]. Moreover, BoNT-A treatment combined with stretching exercises has been shown to enhance overall spasticity management and quality of life [102,103,104].

In conclusion, the evidence from these studies suggests that BoNT-A is a viable and effective treatment option for non-stroke spasticity in conditions such as multiple sclerosis, SCI, and traumatic brain injury. Its ability to improve functional outcomes, pain management, and quality of life, coupled with a favorable safety profile, emphasizes its utility in clinical practice [105]. However, ongoing research, including randomized clinical trials, remains crucial to further establish the efficacy of, and optimize treatment protocols for, BoNT-A in managing non-stroke spasticity.

#### 3.3.4. Efficacy of Botulinum Toxin in Spasticity in Adults

The efficacy of BoNT-A in the treatment of spasticity has been well-documented in various clinical studies published from 2000 to 2022. For upper limb spasticity, trials have consistently shown that AbobotulinumtoxinA, administered at doses ranging from 500 to 1500 U, significantly reduces muscle tone, as evidenced by the Modified Ashworth Scale, with notable yet variable improvements in active movement and pain [106]. Lower limb spasticity studies echo these findings, with AbobotulinumtoxinA demonstrating statistically significant reductions in muscle tone and consistent relief in pain symptoms [107]. Advancements in the treatment of adult spasticity with OnabotulinumtoxinA have been significantly shaped by various clinical trials worldwide. Notably, the REFLEX trial played a crucial role in obtaining FDA approval for the treatment of lower limb spasticity [108,109]. This was complemented by comprehensive studies that further elucidated the efficacy of OnabotulinumtoxinA in managing upper limb spasticity [21,42,110,111]. Two Phase 3 trials were conducted to investigate the use of IncobotulinumtoxinA in treating upper limb spasticity post-stroke. The first trial, including 148 patients, demonstrated sustained improvements in muscle tone and functionality after a single injection and over an extended period with repeated treatments [112]. The second trial involved 259 patients who demonstrated significant improvements in muscle tone and functional disability, with the majority of patients responding positively to treatment [113]. These results further confirmed significant improvements in muscle tone and global impressions of change over three treatment cycles, each 12 weeks apart, with minimal treatment-related adverse events [114]. The TOWER study evaluated the safety and efficacy of increasing doses of IncobotulinumtoxinA in treating patients with limb spasticity due to cerebral causes [50]. Involving 155 patients, the study concluded that increasing IncobotulinumtoxinA doses up to 800 U is safe and tolerable, allowing treatment of a greater number of muscles. Finally, the recent J-PURE phase III double-blind study, which involved participants receiving either 400 U of IncobotulinumtoxinA or a placebo, followed by an open-label extension, demonstrated significant improvements in muscle tone, as measured by the Modified Ashworth Scale [115].

The timing and frequency of repeated botulinum toxin treatments for spasticity are key factors for achieving sustained therapeutic effects. Determining the correct intervals for botulinum toxin treatment in spasticity management is crucial for maximizing the therapeutic benefits. Treatments every 12 weeks up to 56 weeks have demonstrated improvements in muscle tone and quality of life [116]. Longitudinal studies, such as Turner-Stokes et al. [117], have indicated significant upper-limb spasticity and functional improvements over two years with repeated treatments, suggesting maximum efficacy after two to three cycles, especially by week 12 [118]. Research has also explored the efficacy and safety of shorter intervals between injections [119]. In this context, considering molecular pharmacodynamics is essential: it was found that BoNT-A’s maximum effect on muscle spasticity, as measured by changes in the MAS, peaks around 5 weeks post-injection, with variations in effect duration among different formulations. AbobotulinumtoxinA, for instance, maintains its effects for up to approximately 13.1 weeks. This is longer than OnabotulinumtoxinA and others, which last about 8.6 weeks [120]. Moreover, it seems that there is a correlation between the dose and the duration of BoNT-A effect. According to the dose–duration correlation, the amount of BoNT-A administered can impact how long its therapeutic effects last, with higher doses potentially leading to a longer duration of action up to a saturation point near three months, after which additional increases in dose fail to significantly prolong its efficacy [121,122].

Timing of treatment initiation following the onset of spasticity plays a crucial role. While many studies have mainly focused on patients with long-term spasticity (averaging 2.5 years after stroke), it is imperative to discuss the benefits and considerations of early BoNT-A injection (within 3 months of the stroke) [123]. Early treatment refers to initiating medical interventions or therapies as soon as possible after the onset of a disease or condition. This approach focuses on intervening before the spasticity leads to further complications, emphasizing the potential for more effective management and improved outcomes.

Research by Rosales et al. significantly advanced the understanding of timely BoNT-A application in treating spasticity following a stroke, highlighting the therapeutic potential of early intervention for improving patient outcomes [124]. In 2012, they found that early BoNT-A intervention significantly improved function and quality of life in patients with upper limb spasticity [124]. Building on this, their 2016 meta-analysis further confirmed the safety and efficacy of early BoNT-A treatment, emphasizing its crucial role in timely intervention [123]. Lastly, in 2018, Rosales et al. suggested that early treatment with BoNT-A might not only benefit immediate spasticity management but also potentially modify disease progression and reduce the frequency of required re-injections [125]. This theme has been explored further in recent studies [126]. These findings suggest that initiating treatment soon after stroke onset can notably enhance motor re-learning, which is crucial for rehabilitation [127]. Additionally, early intervention is associated with reduced contracture development, without interfering with the recovery of arm function [128]. Moreover, integrating this treatment with multimodal rehabilitation therapies significantly improves functional recovery and quality of life [129].

It is important to note that some limitations may derive from defining early intervention for spasticity as treatment administered within 3 months of the event. The onset of spasticity varies, with some cases emerging 6 months post-stroke. Additionally, data on early treatment for spasticity due to other causes like traumatic brain injury and spinal cord injuries, which often lead to severe complications, are lacking. Therefore, early treatment should be defined by the onset of spasticity symptoms rather than a fixed time since an event, emphasizing the importance of early detection before considering early treatment [130]. Essential to this approach is the early detection of spasticity, emphasizing the need to identify prognostic indicators and predictive markers for the onset of spasticity, especially in its more disabling forms [131,132,133]. In the context of spastic paresis, understanding the effectiveness of early treatment and its impact on function requires not just predictors of spasticity but also predictors of function [134] to distinguish patients who may benefit from early treatment in terms of both spasticity and functionality. Further research is necessary to explore the incidence of spastic paresis and identify predictive markers for this condition.

#### 3.3.5. Safety of Botulinum Toxin Injection in Adults

Botulinum toxin therapy is widely recognized for its safety in long-term treatment of spasticity and muscle contraction-related diseases. It has been consistently proven safe for various clinical applications over the years [21,32,42,51,114,135,136,137]. Adverse events from BoNT-A therapy can include local reactions, unintended muscle weakness, dysphagia, spread of toxin effects, allergic reactions, respiratory problems, flu-like symptoms, and autonomic dysreflexia, and their likelihood and severity are influenced by factors such as dosage, injection site, and patient-specific characteristics. Severe adverse events are rare, thus reinforcing the safety profile [68,138,139,140,141].

The treatment of spasticity with botulinum toxin involves specific dosage guidelines that vary between formulations. According to the FDA, for OnabotulinumtoxinA administration, adults should not exceed a total dose of 400 units within a 3-month interval, while pediatric doses should not surpass the lesser of 10 units/kg or 340 units. IncobotulinumtoxinA is recommended at up to a 400 units total dose for adult upper limb spasticity, divided among affected muscles. For pediatric patients, excluding those with cerebral palsy-induced spasticity, the dosage is 8 units/kg (up to 200 units) for a single upper limb or 16 units/kg (up to 400 units) for both upper limbs. AbobotulinumtoxinA suggests a maximum of 1500 units per session for adults, with specific guidelines for upper and lower limb spasticity at 640 Units and 1000 units, respectively, depending on body weight and treatment extent. European guidelines advise not to exceed 1500 MU for AbobotulinumtoxinA and 600 U for OnabotulinutoxinA per session, with a maximum of 125 MU for AbobotulinumtoxinA and 50 U for OnabotulinumtoxinA per injection site, recommending multiple sites for larger muscles [12]. For pediatric patients, total doses of 400–600 units for OnabotulinumtoxinA and 500–1000 units for AbobotulinumtoxinA were indicated [17]. These dosing guidelines reflect a careful balance between efficacy and safety, underlining the necessity for precise dosing to maximize therapeutic benefits while minimizing the risk of adverse effects.

The relationship between botulinum toxin dosage and the incidence of adverse events is a topic of considerable interest and has been explored in various studies. Bakheit et al. [27] and Pittock et al. [142] set out a randomized, double-blind, placebo-controlled trial assessing the efficacy and safety of three doses of AbobotulinumtoxinA (500, 1000, and 1500 units) in post-stroke spasticity. Bakheit et al. found the optimal dose for upper limb muscle spasticity was 1000 units, emphasizing minimal adverse events and establishing safety parameters, while Pittock et al. demonstrated significant improvements in calf spasticity and limb pain without substantial safety concerns even with higher doses, such as 1500 units. These studies underscore the importance of dose optimization and safety in BoNT-A therapy across different muscle groups.

Subsequently, Dressler et al. focused on the safety aspects of high-dose IncobotulinumtoxinA therapy by comparing high-dose and regular-dose injections in patients with various forms of dystonia and spasticity [143]. The high-dose group received IncobotulinumtoxinA doses significantly above the standard, going up to 1200 units. This study evaluated systemic toxicity and found that high doses of IncobotulinumtoxinA can be administered safely without detectable systemic toxicity. Santamato et al. further explored high-dose IncobotulinumtoxinA in treating post-stroke spasticity. The 2013 study pioneered the investigation of high doses (up to 840 units), finding significant reductions in spasticity and pain with improved disability outcomes, without major adverse events [144]. Building on this, the same group affirmed the effectiveness and safety of these high doses in a 2016 study [145]. In 2017, they further extended these findings, demonstrating the long-term safety and sustained efficacy of repeated high doses over two years [146]. Wissel et al. concluded this progression by safely administering IncobotulinumtoxinA up to 800 units, underscoring the high dosage tolerance in limb spasticity [50]. This series of studies showcases the evolving understanding and application of high-dose IncobotulinumtoxinA therapy in clinical practice.

In contrast to the IncobotulinumtoxinA studies, Baricich et al.’s research on OnabotulinumtoxinA demonstrated that high doses (up to 800 units) effectively managed post-stroke spasticity without adverse events [147]. This parallel narrative was complemented by their 2017 study, which examined the effects of high doses of both IncobotulinumtoxinA and OnabotulinumtoxinA on heart rate variability (HRV) in chronic hemiplegic stroke patients, showing no significant changes in HRV and indicating no adverse effects on the autonomic heart drive [148]. Kirshblum et al. further contributed to this body of knowledge by examining the safety profile of higher (>600 units) and lower-dose botulinum toxin injections [149]. They observed a significant increase in adverse events with doses over 600 units, and no significant difference between OnabotulinumtoxinA and IncobotulinumtoxinA regarding adverse event rates. Documented adverse events were categorized into dysphagia, pain or discomfort, respiratory issues, virus-like syndrome or fatigue, unintended weakness, and unrelated events [149]. This research suggests that while doses up to 600 units are generally safe, exceeding this limit may increase the risk of adverse effects, emphasizing the importance of balancing benefits and risks in high-dose BoNT-A therapy. The implications of these findings are significant for clinical practice, suggesting that practitioners can confidently employ BoNT-A at varying dosages tailored to individual patient needs, without compromising safety. This adaptability enhances the scope of personalized medicine for spasticity management, potentially leading to improved patient outcomes and quality of life. Not only do increased doses elevate the probability of experiencing adverse events, but they also possibly contribute to the development of immune resistance [150]. Furthermore, high dosages of botulinum toxin can impact the costs of therapy significantly. Despite the advantages derived from the utilization of high dosages in spasticity, more studies are warranted to assess whether higher dosages can be managed while taking into account both economic and safety concerns.

In the therapeutic use of BoNT-A for conditions such as spasticity and movement disorders, a crucial consideration is the phenomenon of reduced response or non-response, particularly in the context of long-term treatment. Reduced or non-response can be attributed to inappropriate muscle selection, inaccurate injection placement, insufficient dosage, improper patient selection, lack of specific treatment goals, progression of the underlying disease, or handling errors during drug storage or preparation [151]. Permanent non-response is rare and often attributed to immunological reactions [152]. Primary non-response might occur in patients with reduced sensitivity to botulinum toxin, whereas secondary treatment failure is more likely due to the development of neutralizing antibodies (NAb) against the neurotoxin. In this case, patients initially exhibit a good clinical response and subsequently experience a loss of treatment efficacy because the body’s immune system recognizes the therapeutic protein as a foreign substance, leading to the production of Nab, which diminishes the effectiveness of the drug [153]. The risk of NAb formation and subsequent secondary non-response is influenced by several factors, including the frequency of injections, total dosage administered, and the specific BoNT-A formulation used in treatment.

Carr et al. emphasized the need to consider the immunogenic potential of different BoNT-A formulations, highlighting the risk of neutralizing antibody formation with repeated use [56]. This aspect is crucial, as NAb development can lead to clinical non-responsiveness, thus affecting the long-term success of therapy. Mathevon et al.’s systematic review, encompassing 14 articles, including five randomized controlled trials and various observational studies, revealed that Nab prevalence was approximately 1%, and was consistent across different BoNT-A formulations [55]. This study also found that NAb positivity was favored by long-term therapy, high doses, and short intervals between injections. Comparatively, Hefter et al. showed no NAb development in patients treated with complex protein-free IncobotulinumtoxinA. In contrast, patients treated with complex protein-containing formulations experienced higher rates of NAb development, with 5.9% of those who did not switch preparations and 33.3% of those who switched between different types being affected [154]. The probability of developing NAb, as highlighted by Hefter et al., is highly relevant in clinical practice. It is important to note that the study by Hefter et al. merged data on OnabotulinumtoxinA and AbobotulinumtoxinA into a single group, despite these being distinct formulations with differing potencies. Jankovic et al.’s meta-analysis, spanning 33 clinical trials with nearly 30,000 subject records, found an overall low NAb formation rate of 0.5% following OnabotulinumtoxinA treatment [155]. Importantly, this study showed no clear association between NAb-positive events and higher doses, number of treatment cycles, or other clinical variables. Together, these studies highlight the low but not negligible risk of NAb development in BoNT-A therapy, which is influenced by factors such as formulation, treatment duration, dosage, and frequency of injections. They emphasized the necessity for careful formulation choice and treatment strategy in BoNT-A therapy to ensure sustained patient responsiveness while minimizing the risk of treatment resistance due to NAb development. These findings collectively suggest that while NAb development can impact clinical response, its overall incidence is low and often does not compromise the efficacy of the treatment.

#### 3.3.6. Therapeutic Implications of Adjunctive Therapies and Multimodal Approach in Spasticity Treatment

Adjunctive therapy, employed alongside botulinum toxin injections, plays a crucial role in enhancing the efficacy of spasticity treatments. A variety of adjuvant treatments, including adhesive taping, casting, electrical stimulation, modified constraint-induced movement therapy, physiotherapy, and splinting, have been studied for their potential to improve outcomes following botulinum toxin injections [52,156].

Bibliometric analysis revealed earlier interest in adjunctive treatment (Figure 9). Hesse et al. investigated the combined use of BoNT-A and electrical stimulation in the treatment of chronic upper-limb spasticity post-stroke [39]. The research revealed that the combined approach was more effective than BoNT-A alone, indicating significant improvement in tasks such as hand hygiene and reduction of elbow and wrist spasticity. Corry et al. explored the effectiveness of combined botulinum toxin injections and electrical stimulation in treating upper-limb spasticity after stroke [38]. The results suggested that this combined approach enhances the efficacy of botulinum toxin, showing significant improvements in muscle tone and functional activities of the upper limb. Both studies support the idea that adjunctive treatments can amplify the benefits of botulinum toxin in managing spasticity, providing a more comprehensive treatment strategy.

The ongoing attention dedicated to developing more comprehensive treatment strategies for spasticity management is highlighted by the recent citation burst of a paper by Picelli et al., which provided an extensive review of adjunctive treatments to enhance the effectiveness of BoNT-A in managing spasticity [52]. It discussed various non-pharmacological treatments, such as muscle stretching, taping, casting, splinting, and physical modalities, such as extracorporeal shock wave therapy (ESWT), therapeutic ultrasound, vibration therapy, electrical stimulation, and transcutaneous electrical nerve stimulation. Both casting and adhesive taping were shown to significantly improve the effects of botulinum toxin in managing upper and lower limb spasticity [157]. Allart et al. further explored the effectiveness of various adjunctive therapies alongside botulinum toxin injections for the treatment of spasticity [158]. Their study encompassed joint posture procedures, including both continuous techniques, such as taping and casting, and discontinuous methods, such as splinting and manual stretching. They also explored the use of physical agents, such as electrical stimulation, muscle vibration, and shockwave therapy, but advised against these due to limited efficacy. Additionally, the study considered active adjunct therapies, including high-intensity device-assisted methods, and soft posture techniques, such as compression sleeves and kinesiotaping, although these were found to be less effective [158].

Extracorporeal Shock Wave Therapy (ESWT) has garnered attention as an adjunctive modality in spasticity management, particularly when combined with BoNT-A injections. This combination has been increasingly recognized for its synergistic effects in treating post-stroke spasticity, as well as in conditions such as multiple sclerosis and CP. Santamato et al., in their SBOTE (Spasticity treated by Botulinum Toxin and ESWT) study, highlighted that ESWT may enhance the effects of BoNT-A by modulating muscle rheology and neurotransmission at the neuromuscular junction, suggesting a deeper level of interaction between these therapies [159]. Further evidence published by Mihai et al. underscored the benefits of this combination, indicating significant improvements in spasticity management across various patient groups [160]. Duan et al. specifically noted that the use of BoNT-A with ESWT significantly relieved triceps spasticity, improved motor function, and daily living ability in stroke patients [161]. This finding is particularly compelling as it demonstrates the real-world impact of this combined approach on patient functionality and quality of life. The efficacy of this combination is not limited to adults; it also extends to pediatric care. Kwon et al. reported sustained improvements in controlling spasticity in children with CP when treated with both botulinum toxin A and ESWT [162]. This improvement was observed up to three months post-treatment, indicating the lasting benefits of this therapeutic approach.

Additionally, the integration of newer technologies, such as brain stimulation and robotic treatment, with BoNT-A represents a research frontier in spasticity treatment. For instance, Pennati et al. found that short robotic training combined with botulinum toxin neurolysis effectively reduced spasticity and improved motor function in patients with chronic post-stroke upper limb spasticity, opening avenues for more comprehensive, technology-assisted multimodal treatment strategies [163].

In the context of managing spasticity, there has been a shift from considering adjunctive therapies as mere supplements to botulinum toxin injections towards embracing a multimodal treatment approach [8,164]. Multimodal treatment in spasticity care involves a synergistic combination of physical, pharmacological, and surgical interventions where necessary. This approach is particularly effective in complex cases, such as in children in vegetative and minimally conscious states, where the integrated application of these methods achieves optimal outcomes [164]. The multimodal approach not only enhances the effectiveness of spasticity management but also aligns with the individualized functional needs, goals, and preferences of each patient.

#### 3.3.7. Innovations in Diagnostics and Treatment Evaluation

In spasticity assessment, a multitude of innovative measures have been developed, offering a comprehensive approach to understanding and managing this complex condition [165]. Traditional clinical scales, such as the Ashworth and Modified Ashworth Scales, provide baseline subjective evaluations of muscle tone, but have been scrutinized for their subjective nature and lack of standardization [166,167]. However, the Tardieu Scale offers insights by measuring muscle response to passive stretching at different velocities [168]. Electrophysiological measures, notably electromyography (EMG), are essential for evaluating muscle activity and spasticity by recording the electrical activity of muscles [169]. The advent of medical imaging technologies, including ultrasound and magnetic resonance imaging (MRI), has enabled a detailed view of muscle and tissue changes associated with spasticity. The impact of therapeutic interventions on muscle volume, particularly in the context of managing spasticity through BoNT-A injections, is a critical area of study. Emerging research indicates that while BoNT-A effectively reduces spasticity, its long-term effects on muscle volume and structure warrant thorough investigation [170,171]. Furthermore, Elwischger et al. explored the distribution dynamics of BoNT in muscle tissue, suggesting that injection techniques such as varying injection depths might influence the long-term outcomes of muscle volume and function [172]. These findings underscore the complexity of BoNT-A’s impact, suggesting that while immediate spasticity reduction is evident, the need for refined injection strategies and long-term implications on muscle volume could be further explored.

Ultrasound examination is the most studied technique to explore muscle changes in spasticity [173,174,175]. For instance, an ultrasound-based tool such as the Modified Heckmatt scale has demonstrated good reliability and validity for assessing muscle changes in spasticity, showing a significant relationship with quantitative gray-scale scores [176]. Shear wave elastography (SWE) has emerged as a tool for assessing quantitative muscle stiffness in spasticity [177], particularly for evaluating the therapeutic effects of BoNT-A [178]. Several studies demonstrate the utility of SWE in quantifying changes in muscle stiffness post-BoNT-A treatment [179,180,181]. However, this technique is limited by the need for specialized equipment, variable measurement reliability, and sensitivity to patient positioning and movement [178]. Portable devices and wearable technologies, such as sensors and smart fabrics, have revolutionized the field by allowing real-time, objective data collection on muscle stiffness and movement in everyday settings [182]. Isokinetic dynamometry quantitatively measures muscle strength and spasticity by adding another layer of objective data [183]. Neuromusculoskeletal modeling provides a sophisticated biomechanical perspective for simulating muscle and joint interactions [184]. Additionally, patient-reported outcome measures (PROMs) capture the subjective experience of spasticity, which is crucial for understanding its impact on daily life [185]. Together, these methods encompass a holistic approach to spasticity assessment by blending traditional clinical scales with advanced technology-driven techniques.

Guidance techniques play a crucial role in the administration of botulinum toxin. Various techniques, such as electromyographic guidance (EMG), electrical stimulation (ES), and ultrasound guidance, are employed to improve the efficacy and minimize the adverse effects of these injections. EMG and ES are particularly important for ensuring accurate muscle targeting, thereby enhancing the effectiveness of treatment [186]. Overall, instrumented guidance has been shown to be more effective than manual needle placement in treating spasticity and focal dystonia [187]. By precisely identifying the muscles to be treated, these techniques can contribute to better clinical outcomes and reduce pain, deformity, and caregiver burden [46,188]. Picelli et al. demonstrated that using instrumental guidance, such as electrical stimulation or ultrasonography, for botulinum toxin injections in stroke patients results in better outcomes in muscle tone, spasticity reduction, and joint range of motion compared to manual needle placement [48,189]. Santamato et al. compared ultrasound-guided BoNT-A injections with manual needle placement in patients with stroke and found that the ultrasound-guided method led to significantly better clinical outcomes, including reduced spasticity and improved finger positioning [190]. Ploumis et al. found that using needle EMG guidance for botulinum toxin injections in hemiplegic patients is effective, resulting in greater spasticity reduction and functional improvement compared to injections based on anatomical landmarks [191]. Buyukavci et al. conducted an observational study on post-stroke patients to evaluate the effects of ultrasound-guided BoNT-A injections using the Euro-musculus spasticity approach [192]. They found significant decreases in spasticity and improvements in upper limb motor function, suggesting that this approach is practical and effective for administering injections precisely and improving rehabilitation outcomes [192].

Finally, a recent network meta-analysis revealed that ultrasound-guided BoNT injections are the most effective in treating limb spasticity in adults, followed closely by electrostimulation, electromyography, and manual needle placement, highlighting the overall superiority of guided over non-guided injections [193].

### 3.4. Study Limitations

This bibliometric analysis has several limitations. Primarily, it relies solely on the Web of Science Core Collection (WoSCC) database, potentially omitting pertinent studies from other sources. It included only English-language studies, possibly overlooking significant non-English research. Recent high-quality publications may have been underrepresented because of fewer citations. Although the WoSCC database is regularly updated, citation trends are influenced by time. The analysis focuses on frequency and performance metrics, which do not necessarily reflect the quality or impact of the research. Notably, high citation rates can occur for negative reasons. Self-citations may indicate the potential for overrepresentation of specific authors or research groups. The study’s approach might inadvertently encourage research aimed at higher citation counts rather than advancing knowledge. Furthermore, the automatic cluster labeling of CiteSpace employs algorithms based on the titles of papers, which may not fully capture all variances and comprehensive information in the clusters. An additional limitation is that data from the Web of Science do not distinguish between medical and research institutions, and there is a lack of information on the academic degrees and expertise of authors (e.g., PhD, MD), which could provide valuable context for interpreting the research landscape. Given these constraints, the results should be viewed as an overview of prevailing trends and topics, rather than as a comprehensive depiction of the field. These limitations are partially mitigated by a comprehensive overview in the Discussion section, which attempts to cover the most relevant topics in the field, providing a broader perspective and context for the analysis. To address these limitations, future research could include qualitative content analysis, expert interviews, and stakeholder perspectives for a more complete understanding. As we look ahead, the role of artificial intelligence (AI) in bibliometric analyses becomes increasingly relevant. As AI continues to evolve, its potential application in conducting bibliometric analyses may introduce efficiencies in data processing and pattern recognition. However, the unique value of human expertise in contextual analysis, interpretation, and the synthesis of complex information remains indispensable. Future iterations of bibliometric studies may benefit from AI’s capabilities, yet the critical insights derived from expert analysis will continue to be pivotal in understanding and advancing the field.

## 4. Conclusions and Future Directions

Our comprehensive bibliometric analysis from 2000 to 2022 offers a panoramic view of research relating to the use of BoNT-A in spasticity treatment. This reveals a growing global interest and collaboration in this domain, particularly since 2007, with significant contributions from key countries and institutions. Our study maps the evolution of BoNT-A in spasticity treatment, highlighting the researchers who have shaped the field.

It highlights research hotspots, such as the focus on stroke and cerebral palsy patients as the most studied populations, and underscores the importance of efficacy and safety studies, vital for validating BoNT-A in clinical practice. Emerging trends suggest a potential shift towards earlier intervention post-stroke and integration of botulinum toxin with multimodal rehabilitation strategies. Innovations in diagnostic and treatment evaluation tools such as advanced imaging and wearable technology can offer more precise assessments and treatment outcomes. The integration of guided injection techniques may continue to improve efficacy, reduce adverse events, and enhance patient quality of life for a more patient-centric approach to spasticity management. Additionally, our analysis identifies key research gaps that present opportunities for future research (Table 8). These findings provide a valuable foundation for researchers to build upon in future studies, exploring new frontiers in spasticity management.

## 5. Materials and Methods

### 5.1. Data Collection

**Figure 12:**
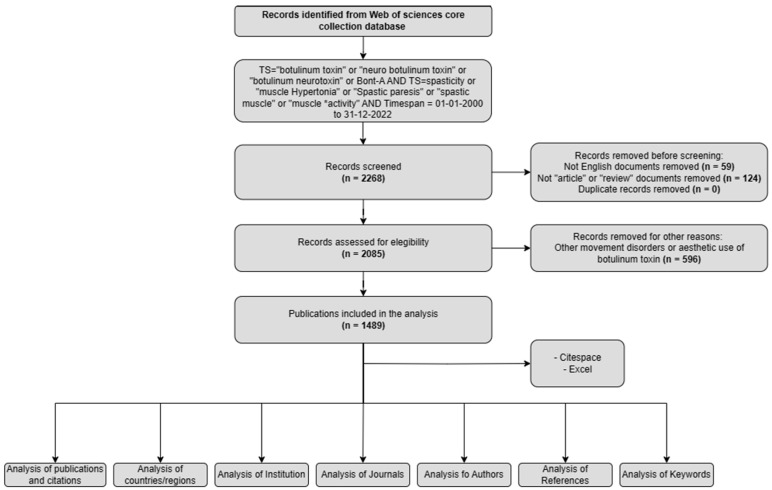
Search strategy and bibliometric analysis framework.

In this bibliometric study, the WoSCC served as the primary data source. The search strategy and data retrieval, detailed in Figure 12, involved an extensive and selective process, focusing on original English articles published between 1 January 2000 and 31 December 2022. A total of 1489 articles were screened, and the analysis was conducted on 30 May 2022. The research was centered around two types of documents, articles and reviews, to provide a broad yet focused view of the field’s empirical and theoretical developments. Exclusions were made for proceedings papers, book chapters, and early access articles to ensure a concentration on formal and substantial scholarly contributions. From the WoSCC database, comprehensive bibliographic details, including publication outputs, research categories, authors, countries, institutions, journals, references, and keywords, were extracted to form the foundation for the subsequent analysis. In the data collection phase, duplicates were analyzed using CiteSpace 6.2.R6. Following the initial data extraction, a manual review process was implemented to refine the dataset. Papers that were not directly relevant to the central theme were systematically excluded. Specifically, studies that discussed alternative applications of botulinum toxin, such as its use in aesthetic treatments or for treating movement disorders other than spasticity, were identified and removed from the dataset. After downloading the references to “.txt” files from WoSCC, two authors conducted an independent review and examined each reference. This process focused on identifying and correcting any inconsistencies in authors’ names, keywords, and other essential bibliographic information. Following the title and abstract review, an additional layer of scrutiny was applied to the top-ranked papers derived from the analysis. These papers were selected based on their prominence and relevance as indicated by the bibliometric analysis. The full texts of the top-ranked papers were then thoroughly read and analyzed.

### 5.2. Data Analysis

The bibliometric and visual analyses were conducted using CiteSpace (version 6.2.R6 Advanced), a software known for its capability in scientometric analysis and visualization [19]. Only for Figure 10 and Figure 5 versions 6.2.R3 and 6.2.R7 were employed respectively. Microsoft Office Excel was used to conduct the R^2^ trend analysis.

This study utilized a comprehensive approach to analyze the relationships and structures within the botulinum toxin research field. Co-occurrence analysis is essential in mapping the connections between words within documents, revealing prevalent themes and patterns [194]. Concurrently, co-citation analysis illuminates the intellectual framework of the field, highlighting significant contributions and emerging trends [195,196]. In the co-citation analysis, self-citations were included to comprehensively understand the development of ideas and the contribution of key researchers within the field. References were systematically divided into two distinct groups for a more granular analysis: the top 10 references from original articles and the top 10 references from reviews. This categorization was applied across two separate periods, the first spanning 2000–2010, and the second spanning 2011–2022. This division not only highlighted the most influential works in each category but also allowed for an analysis that respected the different natures and impacts of articles and reviews within the scientific community.

To enrich the analysis further, this temporal division was instrumental in tracing the developmental trajectory of the field, revealing how key themes, influential research, and pivotal references have shifted and evolved over more than two decades. By dissecting the data in this manner, the study provided a comprehensive and dynamic view of the research trends, key contributions, and intellectual shifts within the field of botulinum toxin research.

Several metrics were employed to delve deeper into the structural aspects of the research network. The average silhouette score provided a measure of the consistency within each cluster [197], the modularity Q index offered insights into the network’s division into distinct modules [198], and betweenness centrality identified how often a particular node appears on the shortest paths between other nodes [199]. In CiteSpace, nodes that have a betweenness centrality greater than 0.1 are highlighted with a purple ring [19]. The cluster structure is significant when Q > 0.3, the clustering result is reasonable when S > 0.5, and persuasive when S > 0.7 [196]. Additionally, burstiness analysis is instrumental in detecting rapid changes in specific research features over time, providing an understanding of the evolving dynamics and impact within the field [200]. In the data collection process, the Impact Factor of journals and authors was considered to assess the influence and prominence of research articles. The Impact Factor for each journal was sourced from the Clarivate Journal Citation Reports (JCR) database on 20 June 2022. The impact factor for the authors was obtained from the Scopus (Elsevier) database on 1 June 2022.

Automatic cluster labeling was performed using the Log-Likelihood Ratio (LLR) and Latent Semantic Indexing (LSI) algorithms within CiteSpace and was based on the titles of the papers [201]. For each type of analysis, specific visualization parameters were set in CiteSpace, as indicated in the figures. This methodological framework is grounded in established bibliometric best practices and guidelines [202].

## Figures and Tables

**Table 1:** The top 10 countries stratified by publication numbers and centrality.

Rank	Country Region	Publications	Country Region	Centrality
1	United States	359	United Kingdom	0.20
2	United Kingdom	170	United States	0.15
3	Italy	169	Italy	0.10
4	Germany	148	France	0.09
5	France	146	Taiwan	0.09
6	Australia	127	Germany	0.08
7	Canada	96	Morocco	0.08
8	South Korea	83	South Korea	0.07
9	Turkey	81	Turkey	0.07
10	Peoples R China	66	Poland	0.07

**Table 2:** Top 10 institutions stratified by publication numbers and centrality.

Rank	Institution	Country	Publications
**1**	Assistance Publique Hopitaux Paris (APHP)	France	68
**2**	UDICE-French Research Universities	France	65
**3**	University of London	United Kingdom	53
**4**	University of Verona	Italy	50
**5**	Universitè Paris Cite	France	42
**6**	University of Texas System	USA	42
**7**	University of Toronto	Canada	42
**8**	University of Foggia	Italy	41
**9**	University of Sydney	Australia	41
**10**	Yonsei University	Korea	36

**Table 3:** Top 10 journals and co-cited journals.

Rank	Journal	P	IF	Co-Cited Journal	Cit	IF
**1**	*Toxins*	81	5.075	*Archives of Physical Medicine and Rehabilitation*	964	4.060
**2**	*Journal of Rehabilitation* *Medicine*	67	3.959	*Neurology*	870	12.258
**3**	*Archives of Physical Medicine and Rehabilitation*	48	4.060	*European Journal of Neurology*	840	6.288
**4**	*Developmental Medicine and Child Neurology*	44	4.864	*Clinical Rehabilitation*	729	2.884
**5**	*American Journal of Physical Medicine Rehabilitation*	43	3.412	*Journal of Neurology, Neurosurgery, and Psychiatry*	701	13.654
**6**	*Disability and Rehabilitation*	40	2.439	*American Journal of Physical Medicine Rehabilitation*	688	3.412
**7**	*Clinical Rehabilitation*	35	2.884	*Developmental Medicine and Child Neurology*	631	4.864
**8**	*PM&R*	33	2.218	*Muscle Nerve*	622	3.852
**9**	*European Journal of Physical and Rehabilitation Medicine*	25	5.313	*Physical Therapy*	598	3.140
**10**	*Frontiers in Neurology*	25	4.086	*Journal of Rehabilitation Medicine*	595	3.959

**Table 4:** The top 15 most productive authors.

Rank	Authors	Country	Institution	Centrality	P	H-Index
1	Santamato Andrea	Italy	Università degli Studi di Foggia	0.02	41	33
2	Picelli Alessandro	Italy	Università degli Studi di Verona	0.07	36	35
3	Wissel Jörg	Germany	Vivantes Klinikum-Spandau,	0.12	34	48
4	Smania Nicola	Italy	Università degli Studi di Verona	0.02	30	51
5	Kanovsky Peter	Czech Republic	Univerzita Palackého v Olomouci	0.05	28	38
6	Turner-Stokes Lynne	United Kingdom	Northwick Park Hospital	0.05	27	47
7	Baricich Alessio	Italy	Azienda Ospedaliera Maggiore della Carita di Novara	0.00	26	23
8	Esquenazi Alberto	USA	Moss Rehabilitation Research Institute	0.03	25	38
9	Gracies Jean Michel	France	Université Paris-Est Créteil	0.01	25	40
10	Bensmail Djamel	France	Université Paris-Saclay	0.04	24	28
11	Ismail Farooq	Canada	University of Toronto	0.00	23	13
12	Ward Anthony B.	United Kingdom	Haywood Community Hospital	0.05	23	34
13	Boulias Chris	Canada	University of Toronto	0.00	22	13
14	Brashear Allison	USA	University of California	0.04	22	40
15	Dressler Dirk	Germany	Hannover Medical School	0.02	22	54

**Table 5:** The top 15 most cited authors.

Rank	Authors	Country	Institution	F	H-Index
1	Simpson David M.	USA	Icahn School of Medicine at Mount Sinai	442	69
2	Bohannon Richard W.	USA	Physical Therapy Consultants	411	73
3	Gracies Jean Michel	France	Université Paris-Est Créteil	350	40
4	Wissel Jörg	Germany	Vivantes Klinikum-Spandau,	333	48
5	Bakheit Abdel Magid O.	United Kingdom	Moseley Hall Hospital, Birmingham	322	26
6	Brashear Allison	USA	University of California	317	40
7	Koman L. Andrew	USA	Wake Forest University Health Sciences	270	38
8	Hesse Stefan	Germany	Medical Park Berlin Humboldtmühle	265	51
9	Esquenazi Alberto	USA	Moss Rehabilitation Research Institute	210	38
10	Bhakta Bipinchandra B.	United Kingdom	University of Leeds, School of Medicine	207	34
11	Rosales Raymond L.	Philippines	University of Santo Tomas Hospital	199	29
12	Dressler Dirk	Germany	Hannover Medical school	198	54
13	Boyd Roslyn N.	Australia	The University of Queensland	195	64
14	Ward Anthony B.	United Kingdom	Haywood Community Hospital	193	34
15	Jancovic Joseph	USA	Baylor College of Medicine	181	157

**Table 6:** The top 10 most cited references 2000–2011.

Rank	Title	Citations	First Author	Journal	Publication Year
1	Spasticity after stroke—Its occurrence and association with motor impairments and activity limitations [20]	460	Sommerfeld, DK	*Stroke*	2004
2	Intramuscular injection of botulinum toxin for the treatment of wrist and finger spasticity after a stroke [21]	418	Brashear, A	*New England Journal Of Medicine*	2002
3	CP [22]	327	Koman, l.	*Lancet*	2004
4	Pathophysiology of spastic paresis. II: Emergence of muscle overactivity [23]	300	Gracies, JM	*Muscle & Nerve*	2005
5	Recommendations for the use of BoNT-A in the management of CP [15]	260	Graham, HK	*Gait & Posture*	2000
6	Assessment: Botulinum neurotoxin for the treatment of spasticity (an evidence-based review)—Report of the Therapeutics and Technology Assessment Subcommittee of the American Academy of Neurology [24]	259	Simpson, DM	*Neurology*	2008
7	Botulinum toxin in clinical practice [25]	256	Jancovic, J	*Journal of Neurology Neurosurgery and Psychiatry*	2004
8	European consensus table on the use of BoNT-A in adult spasticity [12]	246	Wissel, J	*Journal Of Rehabilitation Medicine*	2009
9	Impact of BoNT-A on disability and carer burden due to arm spasticity after stroke: a randomised double blind placebo controlled trial [26]	236	Bhakta, BB	*Journal Of Neurology Neurosurgery And Psychiatry*	2000
10	A randomized, double-blind, placebo-controlled, dose-ranging study to compare the efficacy and safety of three doses of BoNT-A (Dysport) with placebo in upper limb spasticity after stroke [27]	232	Bakheit, AMO	*Stroke*	2000

**Table 7:** The top 10 most cited references 2012–2022.

Rank	Title	Citations	First Author	Journal	Publication Year
1	A systematic review of interventions for children with CP: state of the evidence [28]	757	Novak, I	*Developmental Medicine And Child Neurology*	2013
2	Practice guideline update summary: Botulinum neurotoxin for the treatment of blepharospasm, cervical dystonia, adult spasticity, and headache Report of the Guideline Development Subcommittee of the American Academy of Neurology [29]	297	Simpson, DM	*Neurology*	2016
3	Spasticity after stroke: Physiology, assessment and treatment [30]	221	Thibaut, A	*Brain Injury*	2013
4	Poststroke spasticity Sequelae and burden on stroke survivors and caregivers [31]	150	Zorowitz, RD	*Neurology*	2015
5	Safety and efficacy of AbobotulinumtoxinA for hemiparesis in adults with upper limb spasticity after stroke or traumatic brain injury: a double-blind randomised controlled trial [32]	114	Gracies, JM	*Lancet Neurology*	2015
6	Spasticity After Stroke An Overview of Prevalence, Test Instruments, and Treatments [33]	113	Sommerfeld, DK	*American Journal of Physical Medicine & Rehabilitation*	2012
7	Clinical applications of botulinum toxin [34]	109	Dressler, D	*Current Opinion in Microbiology*	2012
8	Spasticity, Motor Recovery, and Neural Plasticity after Stroke [35]	108	Li, S	*Frontiers in Neurology*	2017
9	New insights into the pathophysiology of post-stroke spasticity [36]	107	Li, S	*Frontiers in Neuroscience*	2015
10	Botulinum toxins: Mechanisms of action, antinociception and clinical applications [37]	106	Wheeler, A	*Toxicology*	2013

**Table 8:** Research gaps in the use of botulinum toxin Type-A in Spasticity.

Category	Research Gap	Description
Clinical Efficacy and Safety	Long-term Efficacy and Safety	More studies are needed on the long-term effects of BoNT-A, especially in pediatric populations and various formulations.
	Comparison Across Formulations	Limited research comparing the effectiveness and side effects of different BoNT-A formulations.
Pharmacology and Treatment approaches	Dose Optimization	Research is required to optimize dosing for different patient groups and conditions. Find the correct Dosage/Timing ratio in different stages of disease and related economic aspects.
	Specific Patient Populations	Need for focused research on BoNT-A’s use in specific populations, such as TBI, SCI, multiple sclerosis, or HSP.
	Early Intervention	Exploration is needed on the role and timing of BoNT-A treatment in early stages of conditions like stroke or cerebral palsy.
Patient-Centered Research	Mechanisms of Action	Further investigation into the molecular and physiological mechanisms of BoNT-A’s therapeutic potential and limitations.
	Development of Resistance	Investigate the development of resistance to BoNT-A, particularly in relation to neutralizing antibodies.
	Quality of Life and Functional Outcomes	Studies focusing on the impact of BoNT-A on quality of life and functional outcomes in different patient populations.
Technology and Multimodal Approaches	Emerging Technologies	Research on integrating new technologies (like robotic therapy or brain stimulation) with BoNT-A treatment.
	Adjunct Therapies and Multimodal Approaches	Detailed studies on the synergistic effects of adjunct therapies and multimodal treatments with BoNT-A are scarce.

BoNT-A: Botulinum Toxin Type-A; TBI: Traumatic Brain Injury; SCI: Spinal Cord Injury; HSP: Hereditary Spastic Paraplegia.

## Data Availability

The data presented in this study are available within the text.

## Supplementary Materials

The following supporting information can be downloaded at: https://www.mdpi.com/article/10.3390/toxins16040184/s1, Table S1: Top 150 Keywords with bibliometric metrics.

## Acronyms

**ASIA**	American Spinal Injury Association
**BONT-A**	Botulinum Toxin type-A
**CP**	Cerebral Palsy
**EMG**	Electromyography
**ES**	Electrical Stimulation
**ESWT**	Extracorporeal Shock Wave Therapy
**FDA**	Food and Drug Administration
**GMFCS**	Gross Motor Function Classification System
**HRV**	Heart Rate Variability
**HSP**	Hereditary Spastic Paraplegia
**IF**	Impact Factor
**JCR**	Journal Citation Reports
**J-PURE**	phase 3 study of upper limb poststroke spasticity in adults from Japan
**LLR**	Log-Likelihood Ratio
**LSI**	Latent Semantic Indexing
**MRI**	Magnetic Resonance Imaging
**Nab**	Neutralizing Antibody
**QOL**	Quality of Life
**REFLEX**	phase 3 study in adult patients with post-stroke lower limb spasticity
**SBOTE**	The Spasticity treated by Botulinum Toxin and ESWT
**SCI**	Spinal Cord Injury
**SWE**	Shear Wave Elastography
**TBI**	Traumatic Brain Injury
**TIM**	Treatment with IncobotulinumtoxinA in Movement
**TIMO**	Treatment with IncobotulinumtoxinA in Movement Open-Label
**TOWER**	Titration Study in Lower and Upper Limb Spasticity
