# Supplementary material for: The Role of Botulinum Toxin Type-A in Spasticity: Research Trends from a Bibliometric Analysis
Source: Toxins (Basel). 2024 Apr 9;16(4):184. doi: 10.3390/toxins16040184 (PMC11053519; doi:10.3390/toxins16040184)
Supplement: Supplementary file 1 [file toxins-16-00184-s001.zip › toxins-2914695-supplementary.pdf]

The Role of Botulinum Toxin Type-A in Spasticity: Research Trends from a Bibliometric Analysis

Table S1. Top 150 Keywords with bibliometric metrics

Freq	Label	Burst	Degree	Centrality	Sigma	Year	ClusterID
599	botulinum toxin	0.00	24	0.04	1.00	2000	2
368	cerebral palsy	0.00	33	0.03	1.00	2000	4
280	stroke	0.00	28	0.04	1.00	2000	2
270	management	16.09	32	0.02	1.47	2000	4
264	spasticity	0.00	33	0.04	1.00	2000	4
259	double blind	18.53	31	0.02	1.33	2000	1
218	children	0.00	40	0.06	1.00	2000	4
217	upper limb spasticity	0.00	51	0.07	1.00	2000	2
207	toxin type a	0.00	41	0.06	1.00	2003	1
196	reliability	0.00	32	0.03	1.00	2000	3
192	efficacy	0.00	31	0.03	1.00	2001	1
191	muscle spasticity	0.00	36	0.04	1.00	2000	3
187	botulinum toxin a	0.00	34	0.06	1.00	2000	2
147	injection	0.00	29	0.06	1.00	2001	2
129	safety	0.00	31	0.03	1.00	2003	1
115	muscle	3.19	44	0.06	1.19	2000	4
114	botulinum toxin type a	5.11	45	0.07	1.38	2000	1
114	placebo controlled trial	9.35	41	0.05	1.64	2000	2
108	injections	7.59	35	0.03	1.26	2000	4
97	therapy	0.00	33	0.03	1.00	2004	4
93	neurotoxin	0.00	31	0.03	1.00	2000	1
89	botulinum toxins	0.00	27	0.03	1.00	2000	2
88	cerebral-palsy	0.00	54	0.12	1.00	2000	0
85	upper extremity	3.71	39	0.05	1.22	2006	3
84	rehabilitation	3.63	26	0.03	1.11	2006	3
80	upper limb	0.00	46	0.07	1.00	2000	3
80	gait	0.00	38	0.04	1.00	2004	4
71	quality of life	0.00	43	0.08	1.00	2004	2
70	trial	10.23	40	0.06	1.61	2000	3
69	randomized controlled trial	5.56	26	0.04	1.22	2003	0
68	modified ashworth scale	0.00	26	0.03	1.00	2003	3
68	poststroke spasticity	6.43	26	0.02	1.12	2008	3
67	adults	6.80	28	0.03	1.22	2003	4
62	gross motor function	0.00	32	0.05	1.00	2003	4
60	scale	0.00	27	0.02	1.00	2007	0
59	stroke patients	3.85	38	0.04	1.16	2002	0
57	limb spasticity	5.03	26	0.02	1.13	2000	1
55	lower limb spasticity	0.00	32	0.04	1.00	2009	0

53	placebo	5.85	33	0.04	1.24	2002	3
52	foot	7.00	37	0.07	1.57	2001	2
51	recovery	0.00	34	0.03	1.00	2003	3
45	ashworth scale	0.00	34	0.04	1.00	2005	3
42	prevalence	7.08	11	0.00	1.03	2008	4
40	multiple sclerosis	0.00	30	0.04	1.00	2006	6
40	botulinum neurotoxin	5.21	27	0.04	1.20	2007	1
39	equinus	0.00	29	0.03	1.00	2002	4
39	intervention	3.23	24	0.02	1.07	2011	4
39	a toxin	15.13	21	0.01	1.21	2000	4
37	disability	0.00	28	0.03	1.00	2003	3
35	electrical stimulation	0.00	45	0.06	1.00	2006	5
35	controlled trial	5.33	31	0.06	1.34	2000	0
35	interrater reliability	0.00	26	0.02	1.00	2010	0
35	manual needle placement	4.87	23	0.01	1.07	2012	0
35	botulinum-toxin	4.22	22	0.02	1.09	2003	6
34	lower limb	3.81	39	0.04	1.14	2004	4
34	pain	0.00	30	0.04	1.00	2006	3
33	upper extremity spasticity	12.32	18	0.01	1.11	2000	2
33	nt 201	5.36	15	0.00	1.02	2013	1
30	electrical-stimulation	5.36	30	0.05	1.27	2001	5
29	gastrocnemius-muscle	7.52	16	0.01	1.05	2011	5
27	impairment	0.00	24	0.02	1.00	2012	3
27	walking	3.20	18	0.01	1.03	2005	4
26	a injections	0.00	44	0.06	1.00	2004	7
26	a injection	0.00	24	0.04	1.00	2004	7
26	adult spasticity	6.05	17	0.02	1.10	2009	0
24	neuromuscular blockade	5.88	32	0.03	1.18	2008	5
24	complexing proteins	5.97	18	0.01	1.06	2009	1
23	traumatic brain injury	3.82	16	0.01	1.05	2004	6
22	spinal cord injury	4.59	13	0.01	1.03	2005	6
21	controlled-trial	5.56	15	0.01	1.04	2011	0
20	induced movement therapy	4.34	30	0.05	1.23	2009	6
20	movement	4.04	29	0.03	1.14	2010	0
20	placebo-controlled trial	5.52	23	0.03	1.15	2000	6
20	classification	5.19	16	0.01	1.07	2012	4
20	pathophysiology	0.00	15	0.02	1.00	2012	5
19	gastrocnemius muscle	3.35	19	0.02	1.05	2012	5
19	onabotulinumtoxina	6.52	13	0.00	1.03	2014	1
18	adverse events	0.00	26	0.02	1.00	2004	4
18	hemiparetic patients	4.03	22	0.01	1.05	2001	2
18	motor function	0.00	14	0.00	1.00	2010	4
17	intramuscular injection	3.60	23	0.02	1.08	2008	5
17	stimulation	0.00	20	0.01	1.00	2014	7
17	validity	3.57	15	0.01	1.04	2008	0
17	shoulder pain	0.00	11	0.01	1.00	2009	3

16	multiple-sclerosis	0.00	21	0.02	1.00	2002	6
16	association	5.45	14	0.01	1.05	2008	3
15	adolescents	0.00	16	0.01	1.00	2015	4
15	tardieu scale	0.00	15	0.01	1.00	2013	0
15	muscles	3.60	13	0.01	1.02	2009	4
15	hemiplegic shoulder pain	4.24	13	0.01	1.06	2010	0
14	botulinum toxin injection	0.00	32	0.06	1.00	2008	0
14	arm	0.00	26	0.02	1.00	2000	3
13	double blind trial	5.73	19	0.04	1.27	2000	7
13	muscle tone	0.00	14	0.01	1.00	2006	0
13	quality-of-life	3.67	14	0.01	1.02	2009	6
13	botulinum toxin-a	0.00	14	0.01	1.00	2010	0
13	guidance	4.30	10	0.00	1.02	2014	5
13	strength	0.00	5	0.00	1.00	2007	5
12	metaanalysis	0.00	23	0.02	1.00	2014	3
12	gait analysis	0.00	20	0.02	1.00	2000	4
12	accuracy	4.37	12	0.00	1.01	2012	5
12	goal attainment	5.00	11	0.01	1.07	2018	3
12	inhibition	0.00	8	0.01	1.00	2005	0
11	neurotoxin type a	0.00	20	0.02	1.00	2006	1
11	term electrical stimulation	3.99	19	0.01	1.06	2005	2
11	ashworth	0.00	17	0.02	1.00	2000	3
11	equinus foot	0.00	14	0.01	1.00	2012	5
11	motor recovery	0.00	11	0.00	1.00	2013	6
11	chronic stroke	0.00	7	0.00	1.00	2015	5
11	upper-limb	0.00	5	0.00	1.00	2010	5
10	localization	0.00	18	0.01	1.00	2009	5
10	botox	0.00	17	0.02	1.00	2003	1
10	modified tardieu scale	0.00	12	0.00	1.00	2014	3
10	international consensus statement	4.33	11	0.00	1.01	2012	4
10	impairments	0.00	11	0.00	1.00	2013	4
10	skeletal muscle	3.43	11	0.01	1.02	2002	5
10	toxin	0.00	8	0.00	1.00	2016	4
10	randomized controlled-trial	3.95	7	0.00	1.01	2009	6
10	abobotulinumtoxina	5.44	3	0.00	1.00	2020	2
9	a treatment	0.00	19	0.03	1.00	2005	0
9	brain-injury	0.00	18	0.02	1.00	2007	3
9	spastic paresis	4.40	13	0.01	1.05	2018	0
9	equinovarus deformity	0.00	13	0.00	1.00	2016	0
9	hemiplegia	0.00	12	0.01	1.00	2006	8
9	medial gastrocnemius	0.00	12	0.01	1.00	2014	5
9	comprehensive rehabilitation	0.00	10	0.00	1.00	2010	7
9	american-academy	3.64	10	0.01	1.03	2010	1
9	injury	0.00	10	0.01	1.00	2002	2
9	limb	4.11	9	0.00	1.00	2002	4
9	systematic review	0.00	7	0.00	1.00	2016	7

9	subcommittee	4.40	5	0.00	1.00	2018	1
8	multicenter	0.00	19	0.01	1.00	2012	1
8	hypertonia	0.00	15	0.02	1.00	2006	4
8	interrater	0.00	10	0.00	1.00	2016	3
8	skeletal-muscle	0.00	9	0.01	1.00	2006	5
8	muscle overactivity	0.00	9	0.00	1.00	2012	6
8	care	0.00	6	0.00	1.00	2014	3
8	reciprocal inhibition	0.00	6	0.00	1.00	2002	5
8	health	0.00	5	0.00	1.00	2014	3
8	type a	4.07	5	0.00	1.00	2015	2
8	american academy	3.91	3	0.00	1.00	2018	1
7	adductor spasticity	0.00	15	0.01	1.00	2008	7
7	activation	0.00	14	0.01	1.00	2006	0
7	botulinum-toxin-a	0.00	13	0.01	1.00	2001	6
7	motor	0.00	12	0.01	1.00	2015	3
7	occupational therapy	0.00	10	1.00	1.00	2008	7
7	dysport(r)	0.00	9	0.00	1.00	2008	7
7	gait velocity	0.00	8	0.00	1.00	2012	0
7	neuropathic pain	0.00	5	0.00	1.00	2012	6
6	deformity	0.00	15	1.00	1.00	2012	0
